# Hypoxia-induced AFAP1L1 regulates pathological neovascularization via the YAP-DLL4-NOTCH axis

**DOI:** 10.1186/s12967-023-04503-x

**Published:** 2023-09-22

**Authors:** Jun-Song Ren, Wen Bai, Jing-Juan Ding, Hui-Min Ge, Su-Yu Wang, Xi Chen, Qin Jiang

**Affiliations:** 1 Department of Ophthalmology, The Affiliated Eye Hospital, Nanjing Medical University, #138 Han-ZhongRoad, Nanjing, 210000 China; 2 The Fourth School of Clinical Medicine, Nanjing Medical University, Nanjing, 210000 China; 3 Department of Ophthalmology, Xuzhou Central Hospital, Xuzhou, 221000 China; 4 Department of Ophthalmology, Northern Jiangsu People’s Hospital, Yangzhou, 225000 China

**Keywords:** Tumor angiogenesis, Ocular pathologic neovascularization, AFAP1L1, Hypoxia, HIF-1α, Vascular tip cell

## Abstract

**Background:**

Pathological neovascularization plays a pivotal role in the onset and progression of tumors and neovascular eye diseases. Despite notable advancements in the development of anti-angiogenic medications that target vascular endothelial growth factor (VEGF) and its receptors (VEGFRs), the occurrence of adverse reactions and drug resistance has somewhat impeded the widespread application of these drugs. Therefore, additional investigations are warranted to explore alternative therapeutic targets. In recent years, owing to the swift advancement of high-throughput sequencing technology, pan-cancer analysis and single-cell sequencing analysis have emerged as pivotal methodologies and focal areas within the domain of omics research, which is of great significance for us to find potential targets related to the regulation of pathological neovascularization.

**Methods:**

Pan-cancer analysis and scRNA-seq data analysis were employed to forecast the association between Actin filament-associated protein 1 like 1 (AFAP1L1) and the development of tumors and endothelial cells. Tumor xenograft model and ocular pathological neovascularization model were constructed as well as Isolectin B4 (IsoB4) staining and immunofluorescence staining were used to assess the effects of AFAP1L1 on the progression of neoplasms and neovascular eye diseases in vivo. Transwell assay, wound scratch assay, tube forming assay, three-dimensional germination assay, and rhodamine-phalloidin staining were used to evaluate the impact of AFAP1L1 on human umbilical vein endothelial cells (HUVECs) function in vitro; Dual luciferase reporting, qRT-PCR and western blot were used to investigate the upstream and downstream mechanisms of pathological neovascularization mediated by AFAP1L1.

**Results:**

Our investigation revealed that AFAP1L1 plays a crucial role in promoting the development of various tumors and demonstrates a strong correlation with endothelial cells. Targeted suppression of AFAP1L1 specifically in endothelial cells in vivo proves effective in inhibiting tumor formation and ocular pathological neovascularization. Mechanistically, AFAP1L1 functions as a hypoxia-related regulatory protein that can be activated by HIF-1α. In vitro experiments demonstrated that reducing AFAP1L1 levels can reverse hypoxia-induced excessive angiogenic capacity in HUVECs. The principal mechanism of angiogenesis inhibition entails the regulation of tip cell behavior through the YAP-DLL4-NOTCH axis.

**Conclusion:**

In conclusion, AFAP1L1, a newly identified hypoxia-related regulatory protein, can be activated by HIF-1α. Inhibiting AFAP1L1 results in the inhibition of angiogenesis by suppressing the germination of endothelial tip cells through the YAP-DLL4-NOTCH axis. This presents a promising therapeutic target to halt the progression of tumors and neovascular eye disease.

**Supplementary Information:**

The online version contains supplementary material available at 10.1186/s12967-023-04503-x.

## Introduction

Angiogenesis is the intricate process of generating fresh blood vessels from pre-existing ones. This process is under the control of specific pro-angiogenic and inhibitory factors, and its state of balance plays a significant role in maintaining tissue growth, repair, and metabolic equilibrium [1]. However, in the occurrence and development of some diseases such as tumor and neovascular eye diseases including age-related macular degeneration (AMD), diabetic retinopathy (DR), corneal neovascularization and retinopathy of prematurity [2], pathological factors such as hypoxia and inflammation will perturb the equilibrium between pro-angiogenic and anti-angiogenic factors. This perturbation leads to anomalous endothelial cell functionality, disordered vascular structure, elevated microvascular density, and augmented permeability, which aim to supply more oxygen, nutrients, and metabolic waste to the abnormal growth of solid tissues [3]. The tumor microenvironment (TME) is a complex and integrated system composed not only of tumor cells but also various types of cells such as endothelial cells, fibroblasts, immune cells, and inflammatory cells, as well as soluble factors such as cytokines and chemokines [4]. The endothelial cells in TME plays a crucial role in the occurrence and development of human cancer, making it a significant research topic in the field. Endothelial cells and their deposited complex extracellular matrix will establish a vascular niche. These endothelial cells not only supply nutrients and O_2_ through the vascular system to facilitate tumor growth, but they also exhibit paracrine secretion, releasing stem and progenitor cell-active trophogens and endothelial-derived growth factors [5]. Remodeling of extracellular matrix in turn regulates the exposure of various angiocrine factors to tumor stem cells and progenitors [6, 7], which together constitute a unique cellular microenvironment that regulates tumor progression, invasion, transport and metastasis. Furthermore, aberrant angiogenesis further contributes to the disruption of the metabolic microenvironment, ultimately intensifying abnormal tumor proliferation and rendering the disease more resistant to chemotherapy, radiotherapy, and immunotherapy [8, 9]. In the last decade, considerable progress has been made in the development of anti-angiogenic drugs that target vascular endothelial growth factor (VEGF) and its receptor (VEGFR). These drugs, such as sorafenib, bevacizumab, leizumab and abebocept, have been extensively utilized in the treatment of tumors or neovascular eye diseases [10–12]. Nevertheless, after repeated treatment with anti-VEGF drugs, a large number of patients develop resistance, and pathological neovascularization continues to grow excessively [13, 14]. Therefore, it is crucial to discover new molecules that regulate angiogenesis and explore their molecular mechanisms in regulating pathological neovascularization. This knowledge can facilitate the development of more selective targeting strategies.

Actin filament-associated protein 1 like 1 (AFAP1L1) is a constituent of the AFAP family, which also includes AFAP1 and AFAP1L2 [15]. These proteins predominantly participate in the control of actin cytoskeletal dynamics, which are indispensable for a diverse range of cellular processes, such as cellular migration, division, and signaling [16–19]. The AFAP family members are believed to exhibit similarities in protein–protein interactions, such as binding to actin filaments and cSrc proteins, due to the highly conserved nature of the domain, the resemblance of the overall sequence, and the likeness of the sequences between SH2/SH3 and PH domains. However, differences in domain sequences also contribute to variations in protein interactions [15, 17]. For instance, cSrc has demonstrated to bind the SH2 and SH3 domains of AFAP1 and AFAP1L2, but with regard to AFAP1L1, the inferred SH3 binding sequence is not entirely inconsistent with the conserved consensus cSrc SH3 binding motif, and are more likely to be a cortactin SH3 domain binding site. Moreover, the varying contents of the three proteins in distinct cells and their distinct subcellular localization determine that their binding molecules participate in different signaling pathways and have significantly different fine biological functions. In recent years, several studies have indicated that AFAP1L1 can participate in the occurrence and progression of tumors, mainly through the regulation of actin cytoskeleton remodeling to regulate tumor cell morphology, movement, and pseudopod formation, thereby affecting tumor cell proliferation, migration, invasion, and epithelial interstitial transformation (EMT) [15, 18, 20–22]. The cytoskeleton is also crucial for the regulation of cell function during angiogenesis [23]. Therefore, it is postulated that AFAP1L1 may represent a novel potential molecule for the regulation of pathological neovascularization.

In this investigation, we ascertained that AFAP1L1 exhibited a linkage with the incidence and progression of numerous tumors and exhibited a significant correlation with the functioning of endothelial cells in the tumor microenvironment via a pan-cancer analysis. Our objective was to scrutinize the function of AFAP1L1 in the pathological formation of new blood vessels and to probe its possibility in the treatment of both neoplasms and neovascular ocular diseases.

## Materials and methods

### Ethics statement

All experiments were sanctioned by the Institutional Animal Care and Use Committee of Nanjing Medical University and performed in accordance with the principles set forth in the Association for Research in Vision and Ophthalmology (ARVO) Statement for the Use of Animals in Ophthalmic and Vision Research. C57BL/6 J, BALB/c Nude and BALB/c (wild-type, WT) mice were procured from the Nanjing Qinglongshan Experimental Animal Center in Nanjing, China.

### Comprehensive pan-cancer analysis

#### Analysis of gene expression

The RNA expression levels of AFAP1L1 in normal tissues were investigated using the Human Protein Atlas (HPA) database (http://www.proteinatlas.org/) [24] and the Genotype-Tissue Expression (GTEx) database (https://gtexportal.org) [25]. In human cancers, the AFAP1L1 expression levels were explored using the "Gene_DE" module in the Tumor Immune Estimation Resource 2 (TIMER2) database (http://timer.cistrome.org/) [26]. Cell type enrichment scores for AFAP1L1 in each tissue was explored using the “Tissue cell type” module in the human protein atlas database (https://www.proteinatlas.org/).

#### Analysis of prognostic potential

The TCGA survival data for AFAP1L1 were investigated using the Tumor Immune Single-cell Hub 2 (TISCH2) database (http://tisch.comp-genomics.org/home/) [27]. The correlation between AFAP1L1 RNA expression levels and pathological stages in various cancers was explored using the “Expression & Stage” module in the Gene Set Cancer Analysis (GSCA) database (http://bioinfo.life.hust.edu.cn/GSCA/#/) [28]. The correlation between AFAP1L1 RNA expression levels and Overall Survival (OS)/Disease Free Survival (RFS) data across various cancers was explored using the “Survival Analysis” module in the Gene Expression Profiling Interactive Analysis 2 (GEPIA 2) database (http://gepia2.cancer-pku.cn/#index) [29].

#### Analysis of DNA methylation

The “Methylation & Expression” module in the GSCA database was utilized to investigate the correlation between AFAP1L1 RNA expression levels and DNA methylation levels across various cancers. The “Methylation & Survival” module was used to examine the correlation between AFAP1L1 DNA methylation levels and Overall Survival/Progression Free Survival (PFS) data across various cancers.

#### Analysis of protein phosphorylation

The phosphorylation sites and levels of AFAP1L1 protein were analyzed between normal tissue and primary tissue based on the Clinical Proteomic Tumor Analysis Consortium (CPTAC) dataset in the UALCAN web portal (http://ualcan.path.uab.edu/) [30, 31].

#### Analysis of immune infiltration

The TCGA data for the infiltration of 22 distinct immune cells was obtained from the TIMER database (http:// timer.cistrome.org/infiltration_estimation_for_tcga.csv.gz) [32], which was then used to examine the correlation between AFAP1L1 expression and immune cell infiltration levels across different cancer types, as ascertained by XCELL algorithms. Additionally, the "Immune" module in the TIMER2 database, a comprehensive tool for analyzing immune infiltration, was used to verify the correlation between AFAP1L1 and infiltration of endothelial cells in diverse cancer types.

#### Analysis of functional enrichment

The top 100 AFAP1L1-related genes were obtained using the "Similar Gene Detection" module from the GEPIA2 database, and then input into the STRING database for display the protein–protein interaction (PPI) network [33]. Subsequently, the version 4.6.2 of the R package ClusterProfiler was employed to conduct Gene Ontology (GO) enrichment analysis on these genes, thereby examining the enriched GO terms. Gene Set Enrichment Analysis (GSEA) is another computational method that determines enriched biological processes based on a pre-ranked gene list defined according to Log2FC values from the limma analysis. The correlation of AFAP1L1 protein between 14 functional states of 41,900 cancer single cells from 25 cancer types were analyzed based on the CancerSEA database (http://biocc.hrbmu.edu.cn/CancerSEA/) [34]. (The abbreviations and corresponding full names of various cancers are shown in Additional file 5: Table S1).

### Processing and analysis of published scRNA-seq Data

The scRNA-seq data originating from the lung endothelial taxonomy database may be utilized for the purpose of analyzing the heterogeneity of lung tumor endothelial cells across various species and models, as well as for the identification of potential angiogenic candidates [35]. To achieve this, the normalized data for the human lung was obtained and subsequently employed to generate a normalized Seurat object using version 4.3.0 of the R package Seurat. Cluster annotation for endothelial cells and tip cells was then performed via expression density analysis of known marker genes in t-SNE projections, utilizing FeaturePlot function in R package Seurat. Data visualization was mainly achieved through the version 3.4.2 of the R package ggplot2. The specific analysis methods employed in pan-cancer analysis and single-cell data analysis were presented in flowchart (Additional file 1: Fig. S1).

### Cell culture and transfection

Human umbilical vein endothelial cells (HUVECs) acquired from American Type Culture Collection was cultured in endothelial cell growth media (ECM) [ScienCell, 1001: FBS, Cat. No. 0025; endothelial cell growth supplement (ECGS, Cat. No. 1052); antibiotic (P/S, Cat. No. 0503)] supplemented with 10% fetal bovine serum (FBS), 100 U/mL each penicillin and streptomycin. A549 cells and Lewis cells acquired from Procell life Science & Technology Co, as a classical lung cancer cell with many characteristics of non-small cell lung cancer (NSCLC), was cultured in 1640 media supplemented with 10% fetal bovine serum (FBS), 100 U/mL each penicillin and streptomycin.

### Plasmid and virus construction

Adeno-associated viruses (AAV) under the control of the endothelial-specific promoter (TIE) and an AAV^ENT^ serotype were purchased from GeneChem Corporation (GeneChem, Shanghai) to induce endothelium-specific knockdown in mouse tumors, retina, choroid and cornea. The AAV containing a short hairpin RNA (shRNA) targeting the AFAP1L1-Mus sequence (AAVENT-TIE- AFAP1L1 shRNA (“AFAP1L1-ECKD #1-#2”)) or scrambled sequence (AAV^ENT^-TIE-NC shRNA (“NC-ECKD”)) was constructed with titers of 1 × 10^12^ virus particles/ml. Plasmid of pEX3-AFAP1L1-3xFlag (OE AFAP1L1) or pEX3-3xFlag (Vector) and virus of AFAP1L1-shRNA (shAFAP1L1), YAP-shRNA (shYAP), DLL4-shRNA (shDLL4) or control shRNA (shNC), were synthesized by GenePharma. In brief, human full-length AFAP1L1 gene was ligated into a pEX-4 (pGCMV/MCS/T2A/EGFP/Neo) vector and human short hairpin RNA (shRNA) oligos targeting AFAP1L1,YAP or DLL4 were ligated into lentivirus (LV). HIF-1α-siRNA (siHIF-1α), HIF-2α-siRNA (siHIF-2α) and control siRNA (siNC) were synthesized by GenePharma. (The target sequence of plasmid and constructed virus are shown in Additional file 6: Table S2.)

### Tumor xenograft model

Xenograft tumor generation was similar to that previously reported in another study [36]. Briefly, recipients for the xenograft experiment were 5-week-old BALB/c Nude mice of the wild-type variety, which were kept in accordance with standard procedures. Each mouse received an inoculation of 5 × 10^6^ A549 cells in 100 µL PBS subcutaneously into the right dorsal region. Once the volume of each tumor reached approximately 100 mm3 ("Day-0"), the tumor-bearing mice were randomized into three groups. Four mice per group were then administered intratumoral injections of AFAP1L1-ECKD #1, AFAP1L1-ECKD #2, or control NC-ECKD daily for 7 days, which were suspended in avalanche in vivo transfection reagent at a concentration of 100 nmol/mL of saline buffer per tumor nodule/a dose of 10 μg nucleic acid in 2 μL reagent in 50 μL of 5% glucose solution/mice. Tumor parameters were recorded, and tumor volumes were calculated using the formula V (volume) = length × width^2^/2 (mm^3^). On "Day-15," the tumor-bearing mice (4 mice/group) were sacrificed, and the tumors were removed for vascular visualization.

### Ocular pathological neovascularization model

The Oxygen Induced Retinopathy (OIR) model [37], Laser-Induced Choroidal Neovascularization (L-CNV) model [38] and Suture Induced Corneal Neovascularization (S-CNV) model [39] are established pathological animal models for retinal, choroidal and corneal neovascularization, respectively. (1) In the OIR model, C57BL/6 J mouse pups at postnatal day 7 (P7) were exposed to 75% oxygen for five days, followed by intravitreally injected with AAV at P12, and then returned to room air (21% oxygen) for another five days. At P17, when maximum retinal neovascularization had occurred, all the retina samples were collected for vascular visualization. (2) In the L-CNV model, six-week-old C57BL/6 J mice were intravitreally injected with AAV, followed by the generation of four laser spots (577-nm wavelength,75 μm spot size, 100 ms duration, 100 mW power) approximately two optic disc diameters from the optic nerve using the Laser System (Quantel Medical; wavelength 577 nm) to induce choroidal neovascularization. At day 7 after laser treatment, when maximum choroidal neovascularization had occurred, all the choroid samples were collected for vascular visualization. (3) In the S-CNV model, six-week-old BALB/c mice were subconjunctivally injected with AAV, followed by the stromal layer 2 mm from the central cornea were placed with three 10–0 nylon sutures (MANI,1406) and each suture extended more than 120° of corneal circumference to obtain a standardized angiogenic response of the three cornea segments. At day 7 after suture treatment, all the cornea samples were collected for vascular visualization by slit lamp (Chongqing Kanghua Ruiming S & T Co, SLE-7E).

### Vascular visualization

To detect the vascular system, tumor sections, or retinal and choroidal flatmounts, they were obstructed and permeabilized for 45 min at 37 ℃ utilizing 5% bovine serum albumin and 1% Triton X-100. This was succeeded by incubation with anti-CD31(Abcam, ab28364,1:200) or biotinylated IsoB4 (Thermo Fisher Scientific, I21413, 1:50) overnight at 4℃ respectively. After rinsing with PBS (tumor sections require the corresponding secondary antibody to be applied), the vascularity was captured through an inverted microscope (Olympus Corp, IX73P1F).

### Luciferase assay

The sequences of transcription factors, namely HIF-1α and HIF-2α, were integrated into the transient expression vector, pCDNA3.1. Subsequently, the promoter region sequences, spanning 2000 base pairs upstream of the AFAP1L1 transcription start site (TSS), were inserted into the upstream of the reporter vector, pGL-SV40-RLuc-△Pro-Luc vector. Additionally, transient expression vector pCDNA3.1, co-expressing HIF-1α and HIF-2α, was utilized in cell experiments. After predicting the binding site of the anoxic response element (HRE), mutants were introduced to construct promoter-mutant plasmids (mHRE). HEK293T cells were transfected with pCDNA3.1 (NC, HIF-1α or HIF-2α) along with the luciferase plasmid driven by wild-type hre promoter (wHRE) or mutated hre promoter (mHRE) in AFAP1L1. Subsequently, the luciferase activity was measured utilizing the Dual-Lumi Luciferase Reporter Gene Assay Kit (Beyotime, Shanghai, China) on the GloMax Luminometer (Promega), in accordance with the manufacturer's instructions. Finally, the data from each luciferase assay were analyzed based on the Renilla/firefly luciferase ratio.

### Transwell assay

Transfected HUVECs at a density of 5 × 10^4^ cells per well were plated onto Extracellular Matrix (ECM) in the upper chambers, which were devoid of Fetal Bovine Serum (FBS), while the lower chambers were loaded with medium containing 10% FBS that acted as a chemoattractant. After incubation for 12 h, HUVECs that had migrated through the pores of the filters were fixed with 100% methanol for 30 min, stained with 0.1% crystal violet (Sigma-Aldrich, 548-62-9) for 5 min, and washed twice with Phosphate-Buffered Saline (PBS) for 5 min each. Subsequently, images were captured using an inverted microscope (Olympus Corp, IX73P1F), and the number of migrated cells was quantified using the Fiji (ImageJ) Cell Counter plugin.

### Wound scratching assay

Transfected HUVECs were seeded into 6-well plates and cultured until they reached 90% confluence. To induce a wound, a mechanical injury was inflicted using a 10 μl pipette tip, which generated a wound width of approximately 1 mm. The cells were then subjected to serum deprivation for 24 h, during which they were cultured in ECM media without FBS. The wound area was subsequently visualized using an inverted microscope (Olympus Corp, IX73P1F) and quantified using the polygon selection function of Fiji (ImageJ).

### Tube formation assay

40 µl thawed BD Matrigel Matrix (Corning,356234) was placed into each well of a 24-well plate and allowed to settle for 30 min. To replicate the process of tube formation, Transfected HUVECs at a density of 5 × 10^4^ cells per well were seeded onto the pre-coated plates and cultured in 1 ml ECM meida supplemented with 10% FBS under the condition of 37 °C with 5% CO2. After 4 h, the formation of tubes was captured via photomicrography using an inverted microscope (Olympus Corp, IX73P1F). The length of the tubes formed was determined using the Angiogenesis Analyzer plugin in Fiji (ImageJ).

### Rhodamine-phalloidin staining

To perform a morphological analysis of transfected HUVECs, staining with Rhodamine-phalloidin was conducted in order to visualize the actin cytoskeleton and filopodia. The HUVECs were fixed with 4% paraformaldehyde (PFA) for 20 min at room temperature and then incubated with Rhodamine-conjugated phalloidin (Abcam, ab235138, 1:1000) for 30 min at room temperature. Subsequently, images were captured using an inverted microscope (Olympus Corp, IX73P1F) and the number and average length of filopodia per cell were determined using the FiloQuant plugin in Fiji (ImageJ).

### Three-dimensional (3D) bead sprouting assay

Transfected HUVECs were detached from the cell culture plates using trypsin–EDTA solution, and subsequently suspended in ECM medium enriched with 10% FBS. Following this, a total of 8 × 10^3^ cells were cautiously mixed with 4 ml of cell culture medium and 1 ml of methocel stock solution, which contained 12 mg of methylcellulose (Aladdin, C104984-250 g). This resulted in the formation of spheroids, which were obtained by placing 25 μl drops of the cell solution onto the lid of a cell culture dish (100 × 20 mm), and then incubating the dish upside-down in a humidified cell culture incubator for 24 h. The hanging drops were then carefully washed off using phosphate-buffered saline. Subsequently, the HUVECs were resuspended in 2 ml of methocel solution containing FBS, and 1 ml each of PBS and rat collagen I (R&D system, Shanghai, China) was added gently. The resulting spheroid-collagen solution was added to 24-well plates (1 ml per well) for 24 h. The sprouts were captured using an inverted microscope (Olympus Corp, IX73P1F) and the number and average length of each sprout per cytosphere were determined using the Sprout Morphology plugin in Fiji (ImageJ).

### Quantitative real-time PCR (qRT-PCR)

The entire cellular mRNA was isolated through the utilization of TRIzol reagent (Invitrogen, A33250) by employing the chloroform layering technique, followed by isopropanol precipitation. Subsequently, the isolated mRNA underwent reverse transcription into complementary DNA (cDNA) using the HiScript III RT SuperMix for qPCR (+ gDNA wiper) Kit (Vazyme, A211). Quantitative real-time PCR (qRT-PCR) was carried out utilizing the PowerUPTM SYBRTM Green Master Mix (Applied Biosystems, A25742) on a PikoReal 96 Real-Time PCR System (Thermo Fisher Scientific, TCR0096). The specifically designed PCR primer sequences were as follows: human AFAP1L1 Forward Primer 5'- TGAACACAGCAGACCTCCAC-3' and Reverse Primer 5'- CCGAAGGTCACTCAGGTCAC-3'; human ACTB Forward Primer 5'-ATTCCTATGTGGGCGACGAG-3' and Reverse Primer 5'- TCTCCATGTCGTCCCAGTTG-3'.

### Western blot

The entire protein content was isolated from cells utilizing RIPA lysis buffer (Beyotime, P0013B), enriched with a mixture of protease inhibitors (Roche, 11,697,498,001). Subsequently, the lysates were subjected to centrifugation at 12,000 revolutions per minute for 30 min at 4 °C and the supernatant was collected. Nuclear/cytoplasmic protein fractionation was performed as described below. The protein concentration was ascertained using Pierce™ BCA Protein Assay Kit (Thermo Fisher Scientific, 23,225), following the manufacturer's prescribed procedures. Subsequently, equal quantities of protein samples were mixed with one-fourth the amount of 5 × SDS-PAGE sample loading buffer (Beyotime, p0015) and heated for 10 min at 100 °C to allow for protein denaturation. The protein mixture was then loaded onto the gel and separated by SDS-PAGE. It was later transferred to a polyvinylidene difluoride membrane (Millipore Sigma, IPVH00010), with a pore size of 0.45 µm, and blocked for 2 h at room temperature in TBST-5% milk. After the membranes were incubated with the appropriate primary antibodies followed by secondary antibodies, the target protein bands were visualized using an Enhanced Chemiluminescence kit (Thermo Fisher Scientific, 32,132).

### Antibodies

The following primary antibodies were used: mouse anti-AFAP1L1 (Santa Cruz Biotechnology, sc-376700; IF 1:50, WB 1:1000), mouse DLL4 Antibody (R&D Systems, AF1389; IF 1:200), rabbit anti-CD31(Abcam, ab28364,1:200), mouse anti-DLL4 (Santa Cruz Biotechnology, sc-365429; WB 1:1000), rabbit anti-NICD (Cell Signaling Technology, 4380; WB 1:1000), rabbit anti-HES1 (Abcam, ab71559; WB 1:1000), rabbit anti-HEY1 (Abcam, ab154077; WB 1:1000), rabbit anti-YAP (Cell Signaling Technology, 14,074; IF 1:200, WB 1:1000), mouse anti-β‐actin (Millipore, A5441; WB 1:1000). The following secondary antibodies were used: goat anti-mouse HRP-conjugated antibodies (Beyotime, A0216; 1:1000) and goat anti-rabbit HRP-conjugated antibodies (Beyotime, A0208; 1:1000) were applied for WB, goat anti-mouse IgG (H + L) Highly Cross-Adsorbed Secondary Antibody, Alexa Fluor™ Plus 488 (Abcam, ab150077; IF 1:400), and DyLight 405-AffiniPure Donkey Anti-Goat IgG (H + L) (Jackson ImmunoResearch, 705–475-147,1:400) was applied for immunofluorescence.

### Nuclear/Cytoplasmic protein fractionation

The separation of cytoplasmic and nuclear fractions was performed using a subcellular protein fractionation kit (Thermo Scientific, 87,790). For the cytoplasmic fractionation, cells were lysed with cytoplasmic extraction buffer (CEB) for 30 min at a temperature of 4 °C, and the supernatant (cytoplasmic extract) was collected by centrifugation at 12,000 revolutions per minute (rpm) for 30 min. For the nuclear fractionation, the centrifugal precipitate was suspended and incubated in nuclear extraction buffer (NEB) for 30 min at a temperature of 4 °C, and the supernatant (nuclear extract) was collected by centrifugation at 12,000 rpm for 30 min. In this study, HUVECs were harvested in order to isolate the cytoplasm and nucleus, and the cytoplasmic and nuclear expression of YAP were analyzed via immunoblotting to determine YAP nucleocytoplasmic shuttling.

### Statistics

The statistical analysis was performed using the R software package. For comparisons between two groups, the two-tailed Student's t-test or the Mann–Whitney U test was used. Differences among more than two groups were analyzed using one-way ANOVA with Bonferroni's post hoc test or Benjamin-Hochberg procedure. All experiments were conducted at least in triplicate. A P-value of less than 0.05 was considered statistically significant.

## Results

### AFAP1L1 Expression Levels in Normal Tissues and Various Human Cancers

**Fig. 1:**
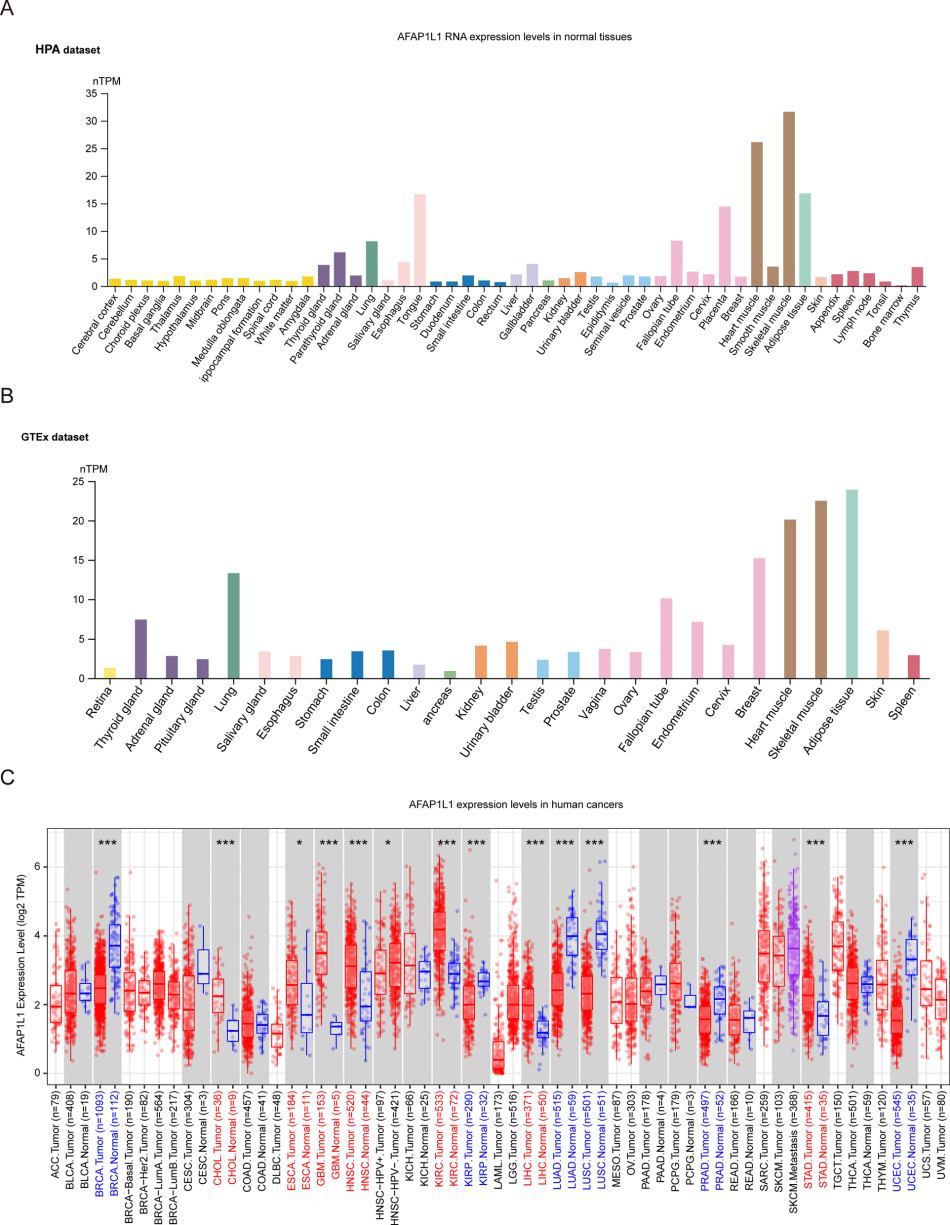
The pan-cancer landscape of AFAP1L1 expression. **A** The mRNA expression of AFAP1L1 in different human tissues from the HPA dataset. **B** The mRNA expression of AFAP1L1 in different human tissues from the GTEx dataset. **C** The mRNA expression of AFAP1L1 in various cancers and normal tissues from the TIMER2 database. Columns labelled with colors indicate statistically significant differences between tumor tissues and normal tissues (upregulation is indicated in red; downregulation is indicated in blue; *: p-value < 0.05; **: p-value < 0.01; ***: p-value < 0.001)

The Human Protein Atlas (HPA) and Genotype-Tissue Expression (GTEx) databases were utilized to examine the expression of AFAP1L1 mRNA in normal tissues. The mRNA expression of AFAP1L1 was predominantly observed in skeletal muscle, heart muscle, adipose tissue, tongue, placenta, and lung in the HPA dataset (Fig. 1A). In addition, the GTEx dataset demonstrated that the primary pattern of AFAP1L1 mRNA expression was consistent with that of the HPA dataset, although it was also highly expressed in breast and fallopian tube (Fig. 1B). Tumor Immune Estimation Resource 2 (TIMER2) database was used to investigate the AFAP1L1 mRNA expression in human cancers. Differential mRNA expression of AFAP1L1 between tumor tissues and adjacent normal tissues was compared, revealing that AFAP1L1 was aberrantly expressed in most human cancer types (Fig. 1C). In particular, AFAP1L1 was significantly up-regulated in various cancer types such as CHOL, ESCA, GBM, HNSC, KIRC, LIHC, and STAD, and significantly down-regulated in LUAD, LUSC, BRCA, KIRP, PRAD, and UCES (Fig. 1C).

### AFAP1L1 Expression Levels is associated with the Prognosis of Various Tumors

**Fig. 2:**
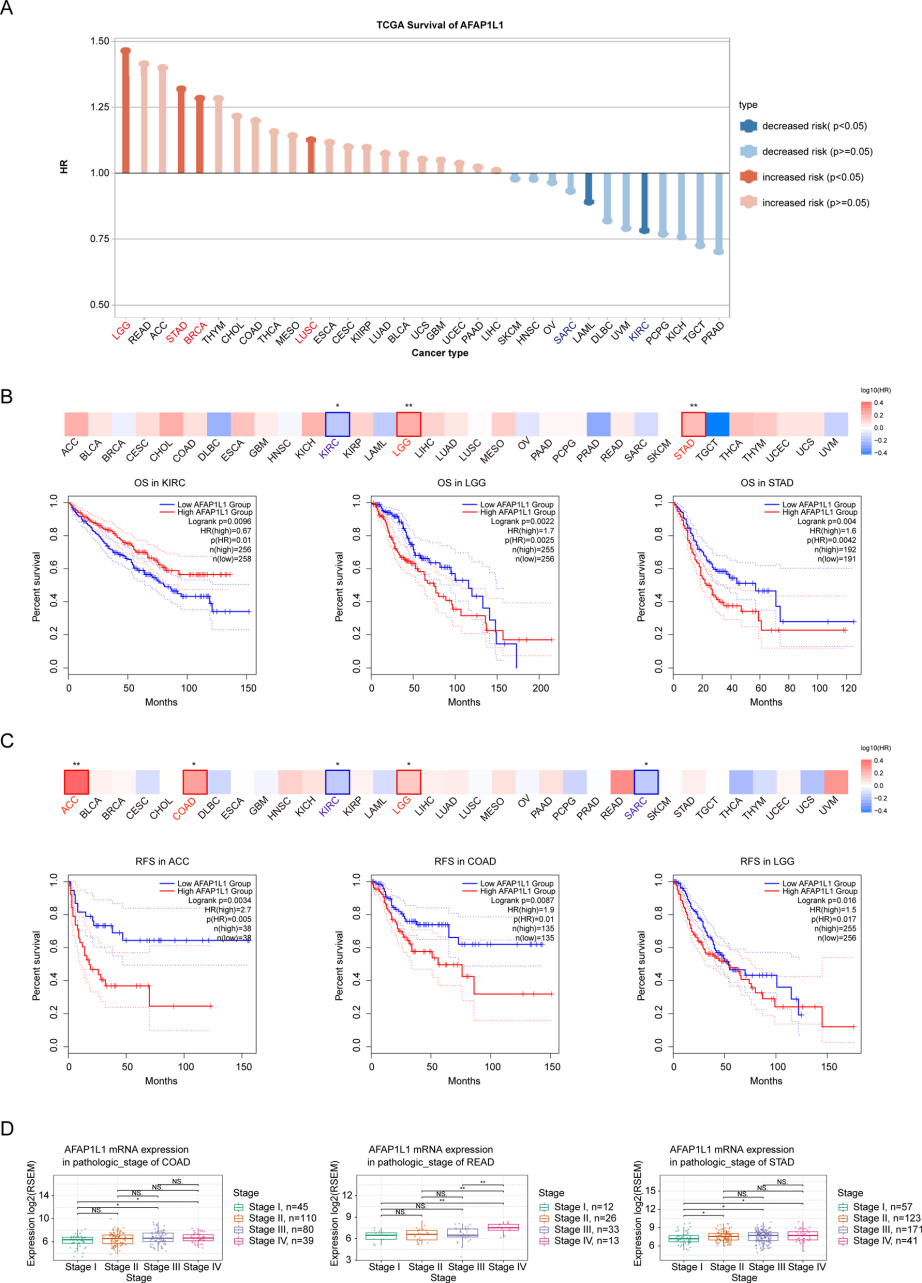
Association of AFAP1L1 mRNA expression with prognosis of various tumors. **A** The TCGA survival data for AFAP1L1 from the TISCH2 database. **B**, **C** The survival curves for OS and RFS manifested that the association between the expression of AFAP1L1 and survival prognosis of pan-cancer patients from the GSCA database. **D** The mRNA expression of AFAP1L1 in pathological stages across human cancers, including COAD, READ and STAD

The Tumor Immune Single-cell Hub 2 (TISCH2) database was employed to examine the TCGA survival data for AFAP1L1. The bar chart indicated that augmented expression of AFAP1L1 significantly amplified the risk of LGG, STAD, BRCA, and LUSC, and concomitantly diminished the risk of SARC and KIRC (Fig. 2A). Furthermore, we appraised the association between the level of AFAP1L1 expression and survival prognosis of pan-cancer patients by means of the Cox regression analysis using the Gene Set Cancer Analysis (GSCA) database. Based on AFAP1L1 mRNA expression level, the survival curves for overall survival (OS) manifested that high expression of AFAP1L1 resulted in worse OS in LGG and STAD patients (Fig. 2B), and the survival curves for disease-free survival (RFS) demonstrated that high expression of AFAP1L1 led to worse RFS in ACC, COAD, and LGG patients (Fig. 2C). An examination of gene expression profiles across multiple human cancer types by using the Gene Set Cancer Analysis (GSCA) database has demonstrated that the expression level of the AFAP1L1 gene was related to the pathological stage of cancer patients. The mRNA expression of AFAP1L1 gene is up-regulated in the late stage of most human cancer types, including COAD, READ, and STAD (Fig. 2D). These findings suggest that AFAP1L1 expression is highly associated with the prognosis of various cancer patients.

### DNA Methylation Analysis and Protein Phosphorylation Analysis of AFAP1L1

**Fig. 3:**
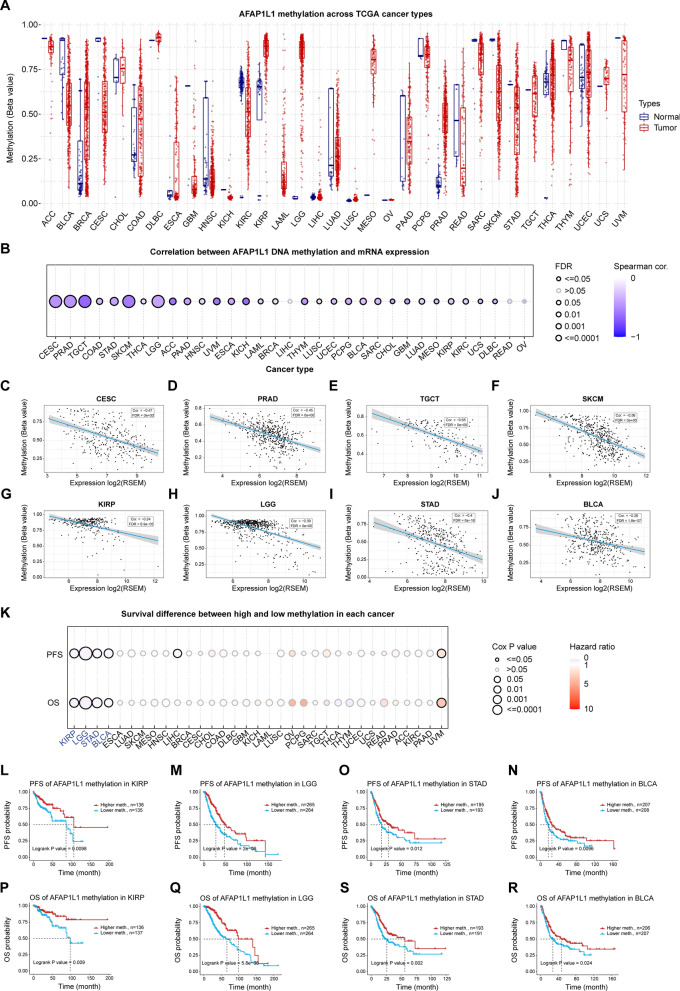
Analysis of DNA methylation of AFAP1L1 in human cancers. **A** The DNA Methylation of AFAP1L1 in various cancers and normal tissues from the GSCA database. **B** Correlation between AFAP1L1 DNA methylation and mRNA expression levels in human cancers from the GSCA database. Several representative correlations were shown as scatter plots, such as **C** CESC, **D** PRAD, **E** TGCT, **F** SKCM, **G** KIRP, **H** LGG, **I** STAD and **J** BLCA. **K** Survival difference between high and low methylation in each cancer from the GSCA database. Several representative correlations were labelled with blue boxes and shown as the survival curves for OS and RFS, including **L**, **P** KIRP, **M**, **Q** ACC, **O**, **S** STAD and **N**, **R** BLCA

It is widely recognized that alterations in DNA methylation can impact gene expression by modifying chromatin structure or transcriptional efficiency. Commonly, DNA methylation is linked to the suppression of gene expression, predominantly achieved through the impediment of DNA-binding proteins that function as either agents or facilitators for transcriptional activation. Alternatively, it involves the recruitment of methyl-binding proteins (MBPs) that can recruit transcriptional co-suppressor complexes, leading to the inhibition of the transcriptional vigor of genes ultimately [40]. Nevertheless, several studies have also revealed that many actively transcribed genes have high levels of DNA methylation in the gene body, which also enriches the role of DNA methylation in gene regulation [41]. Methylation analysis performed by the GSCA has revealed that AFAP1L1 methylation levels are significantly elevated in various types of cancer and normal tissues, such as BRCA, CHOL, COAD, KIRP, LGG, LUAD, PRAD, THCA, and UCES. Conversely, AFAP1L1 methylation levels were found to be diminished in BLCA, CESC, KIRC, and READ (Fig. 3A). Subsequently, we examined the association between DNA methylation and AFAP1L1 expression, and the GSCA results indicated an inverse relationship between them across most cancer types. Specifically, high methylation levels of AFAP1L1 resulted in a reduction in its expression (Fig. 3B–G). Furthermore, based on the DNA methylation status of AFAP1L1, the survival curves for both OS and PFS revealed that elevated methylation of AFAP1L1 led to a worse prognosis in KIRP (Fig. 3K, L, P), LGG (Fig. 3K, M, Q), BLCA (Fig. 3K, N, R), and STAD (Fig. 3K, O, S) patients, with only improved outcomes observed in UVM (Fig. 3K) patients. These findings suggest hypermethylation of AFAP1L1 may increase the risk of poor prognosis in cancer patients.

Furthermore, phosphorylation, a pivotal type of post-translational modification, constitutes a critical molecular mechanism underlying the activation of most proteins, ultimately influencing the initiation and progression of cancer. The CPTAC database was used to analyze differences in AFAP1L1 protein phosphorylation levels between normal and primary tumor tissues. This analysis revealed that AFAP1L1 protein phosphorylation levels were significantly altered at seven sites (S747, S745, S329, S98, S712, S296, and S714) in six types of cancer, including GBM (Additional file 2: Fig. S2A–D), LUAD (Additional file 2: Fig. S2E–G), HCC (Additional file 2: Fig. S2H–J), ovarian cancer (Additional file 2: Fig. S2K–M), clear cell RCC (Additional file 2: Fig. S2N–Q), and PAAD (Additional file 2: Fig. S2R–T). These findings suggest that site-specific phosphorylation of AFAP1L1 may play a potential role in tumorigenesis.

### AFAP1L1 is highly associated with Endothelial cells, especially the Vascular Tip cells

**Fig. 4:**
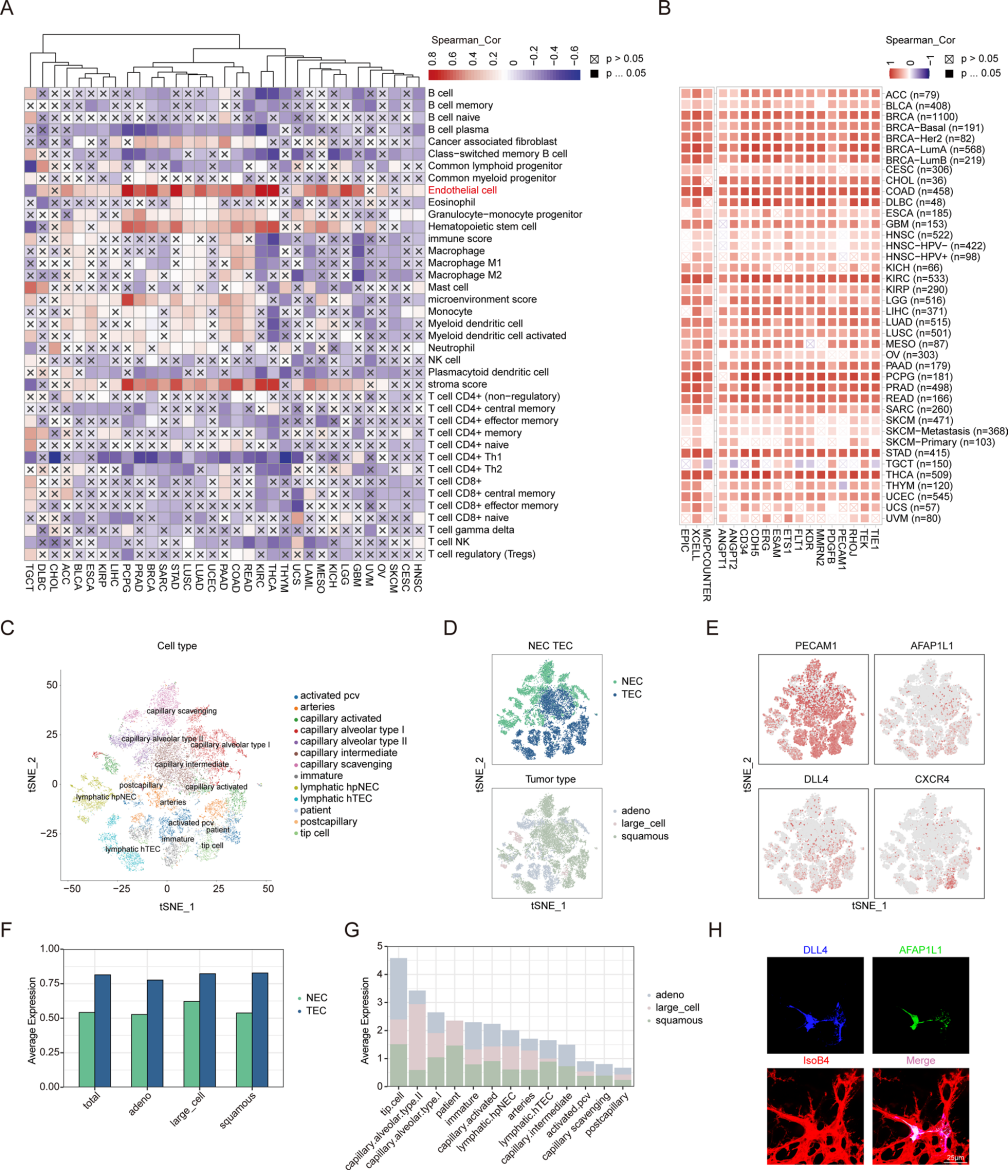
AFAP1L1 is associated with vascular endothelial features in cancer and retina. **A** The heatmap represents the correlation of AFAP1L1 expression with immune infiltration level in diverse cancer types. **B** The heatmap represents the correlation between AFAP1L1 expression level and immune infiltration of endothelial cells and several known vascular endothelial cell markers in diverse cancer types using the TIMER2 database based on EPIC, MCPCOUNTER and XCELL algorithms. **C**–**D** Clustering is performed by the cell type (**C**), the patient types or the tumor types **D** and visualized using Tsne algorithm from the publicly available scRNA-seq data of lung tumor endothelial cell through the Seruat package. **E** Cross validation of the identified EC marker genes (PECAM1), tip cell marker genes (DLL4 and CXCR4) and AFAP1L1 from the publicly available scRNA-seq data of lung tumor endothelial cell through the Seruat package. **F** The grouped bar charts show the expression of AFAP1L1 in total and different types of lung cancer. **G** The stacked bar charts show the expression of AFAP1L1 in different lung cancer types and different cell types. **H** Colocalization of AFAP1L1 and tip cell marker (DLL4) (DLL4: blue; AFAP1L1: green; IsoB4: red) in the anterior end of the retinal vascular. Scale bar: 25 μm. (n = 4 independent experiments)

By utilizing the "tissue cell types" module of HPA database, we have assessed the enrichment score of AFAP1L1 in diverse cell types present in each organization. The findings indicate an elevated expression of AFAP1L1 in endothelial cells across numerous tissues, notably in the Colon (Additional file 3: Fig. S3A, D), Kidney (Additional file 3: Fig. S3A, F), Liver (Additional file 3: Fig. S3A, G), Lung (Additional file 3: Fig. S3A, H), Pancreas (Additional file 3: S3A, I), Prostate (Additional file 3: Fig. S3A, J) and Thyroid (Additional file 3: Fig. S3A, M). Current research indicates that the development of tumors is influenced by not only the tumor cells but also the tumor microenvironment (TME), consisting of endothelial cells, fibroblasts, immune cells, and other factors, that plays a critical role in the onset, progression, and metastasis of human cancer [4]. Due to the robust expression of AFAP1L1 in endothelial cells within numerous normal tissues, we sought to further investigate whether AFAP1L1 is associated with endothelial cells within the tumor microenvironment. We obtained TCGA data for the infiltration of various immune cells from the TIMER database to examine the correlation between AFAP1L1 expression and the levels of tumor-infiltrating immune cells across diverse cancer types. Interestingly, the results demonstrated that AFAP1L1 expression exhibited a significant and positive correlation with endothelial cells, hematopoietic stem cells, and stroma scores in most cancer types within the TCGA database. Notably, in the endothelial cell lines, moderate or strong correlations (r > 0.3) were observed in 21 cancer types, specifically ACC, BLCA, PCPG, PRAD, BRCA, SARC, KIRC, STAD, LUSC, LUAD, UCEC, PAAD, COAD, READ, KIRC, THCA, MESO, KICH, LGG, GBM and OV (Fig. 4A). Similar results were obtained from the TIMER2 database using EPIC, MCPCOUNTER, and XCELL algorithms to examine the association between AFAP1L1 expression and endothelial cells within various cancers (Fig. 4B). To acquire additional evidence supporting the association between AFAP1L1 and endothelial cells, we selected specific markers (ANGPT1, ANGPT2, CD34, CDH5, ERG, ESAM, ETS1, FLT1, KDR, MMRN2, PDGFB, PECAM1, RHOJ, TEK and TIE1) for correlation analysis within the TIMER2 database. The outcomes indicated a significant positive correlation between AFAP1L1 and endothelial cells (Fig. 4B), suggesting that AFAP1L1 may be phenotypically similar to an endothelial cell specific marker.

To elucidate the association between AFAP1L1 and vascular endothelial cells, we procured normalized data from the RNA-Seq lung endothelial classification database. This data enabled us to examine the heterogeneity of lung tumor endothelial cells in various species and models, and recognize potential angiogenic candidates. We employed the R package Seurat to produce normalized Seurat objects, which we then used to reanalyze the aforementioned data. Subsequently, we performed cluster analysis of endothelial cells in lung cancer, and identified 13 clusters in a two-dimensional t-SNE (Fig. 4C). We re-annotated the t-SNE projection according to 'NEC TEC' and ‘tumor type’ to analyze cell distribution in different disease states (Fig. 4D). To obtain a more comprehensive understanding of the expression of AFAP1L1 in lung cancer endothelial cells, we assessed the expression density of well-known endothelial cell marker genes (PECAM1), tip cell marker genes (DLL4 and CXCR4) and AFAP1L1 in the t-SNE projection. The findings revealed that AFAP1L1 was highly expressed in tip cells (Fig. 4E). The histogram indicated that AFAP1L1 was significantly expressed in all three types of lung cancer (adeno, large_cell, and squamous), as well as in total lung cancer patients (Fig. 4F), with the highest expression observed in tip cells (Fig. 4G). This suggests that AFAP1L1 may be highly associated with endothelial tip cells. Considering the surface distribution of the retinal capillary network, the developing retina provides a suitable platform to visualize tip cells. Therefore, we extracted retinas of mice at postnatal day 6 to investigate whether AFAP1L1 was expressed in endothelial tip cells at the leading edge of retinal blood vessels. The immunofluorescence results showed that AFAP1L1 was colocalized with endothelial tip cell markers (Fig. 4H). These results suggest that AFAP1L1 is highly associated with endothelial tip cells under physiological or pathological conditions, and may affect their function.

### Pan-Cancer Functional Enrichment Analysis of AFAP1L1

**Fig. 5:**
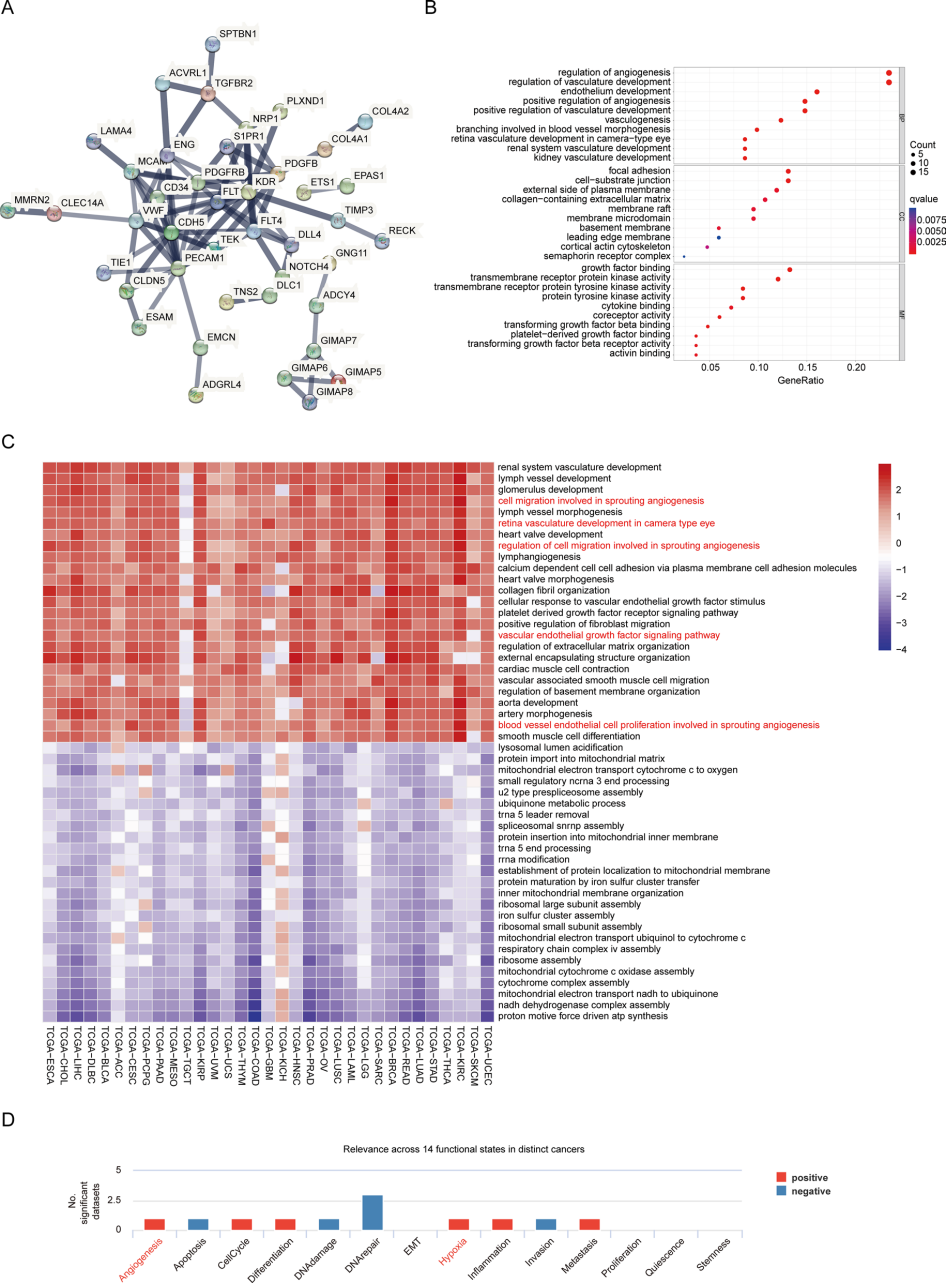
AFAP1L1-related gene enrichment analysis. **A** STRING protein network map of top 100 AFAP1L1-related genes from the GEPIA2 database. **B** GO analysis based on the top 100 AFAP1L1-related genes. **C** According to the differentially expressed genes between the high and low expression groups of AFAP1L1, GSEA analysis revealed the top 25 up-regulated and the top 25 down-regulated enrichment biological processes. Enrichment biological processes ranked based on the normalized enrichment scores (NES) are shown in the heat map. **D** Average correlations between AFAP1L1 and 14 functional states in different cancers from the CancerSEA database and the bar charts show the number of datasets in which AFAP1L1 is significantly related to the corresponding state

In order to investigate the function and possible molecular mechanism of AFAP1L1 in oncogenesis and development, we identified the top 100 AFAP1L1 related genes in the GEPIA2 database. Related genes with high confidence (minimum required interaction score > 0.7) were selected through the STRING database for display PPI network (Fig. 5A). Next, we performed GO functional enrichment analysis for these genes and obtained the first ten terms. GO results showed that most of these genes were involved in and promoted angiogenesis and vasculature development, branching involved in blood vessel morphogenesis and retina vasculature development in camera − type eye (Fig. 5B). In addition, we used GSEA to find that AFAP1L1 was associated with multiple biological processes for 33 cancer types in the TCGA database. GSEA results showed significant positive enrichment in cell migration involved in sprouting angiogenesis, retina vasculature development in camera type eye, regulation of cell migration involved in sprouting angiogenesis, vascular endothelial growth factor signaling pathway and blood vessel endothelial cell proliferation involved in sprouting angiogenesis (Fig. 5C). It is well known that vascular endothelial tip cells play an important role in sprouting angiogenesis. Therefore, combined with these results, we infer that AFAP1L1 may be involved in angiogenesis by regulating the behavior of tip cells. Analysis of associations between AFAP1L1 and 14 functional states in different cancers from the CancerSEA database showed that AFAP1L1 was positively associated with angiogenesis across multiple data sets, what attracts our attention is that it is also positively correlated with hypoxia (Fig. 5D).

### AFAP1L1 regulates tumorigenesis in vivo by regulating tumor vascular structure

**Fig. 6:**
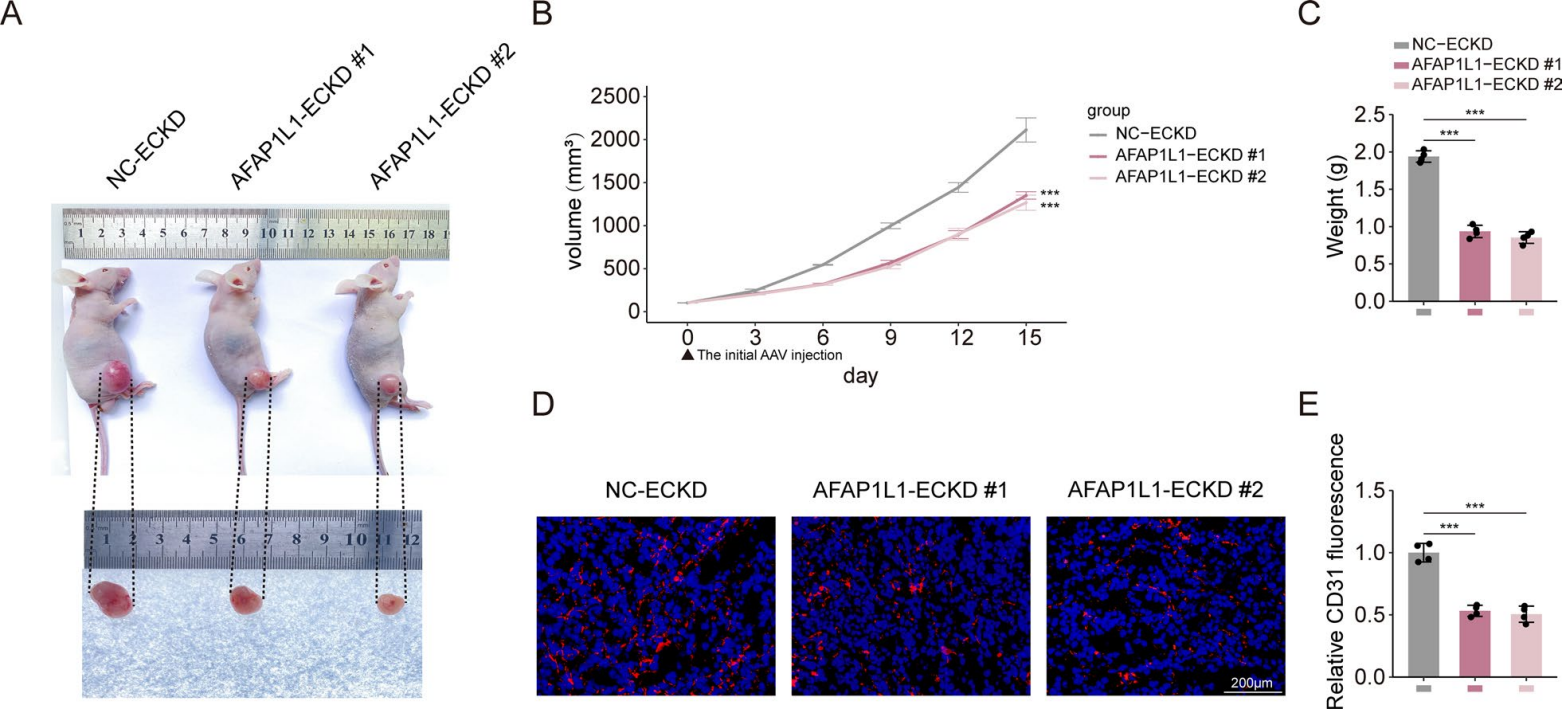
AFAP1L1 knockdown promotes tumor growth and angiogenesis in vivo. **A–C** Xenografts were established by injecting A549 cells subcutaneously on the dorsal flank of BALB/c nude mice, and intratumoral administration of NC-ECKD, AFAP1L1-ECKD #1 or AFAP1L1-ECKD #2 was performed once the tumor length reached 100 mm.^3^ (n = 4 mice for each group). Representative images were shown (**A**); quantification of tumor growth curves of xenograft in nude mice (Day 0 represents the time of the initial AAV injection) (**B**); quantification of tumor weights of xenograft in nude mice (**C**). **D**–**E** Xenografts at the end points were sliced into sections for vascular visualization (n = 4 mice for each group); the sections were labeled with IsoB4 (red) and the nuclei stained with DAPI (blue) (**D**); quantification of IsoB4 stained vessels in the sections of xenografts (**E**). P values were calculated by one-way ANOVA with Bonferroni’s post hoc test (**B**, **C**, **E**). Error bars represent the mean ± SD. *, p < 0.05; **, p < 0.01; ***, p < 0.001

As a crucial component of the tumor microenvironment, endothelial cells facilitate pathological neovascularization, thereby augmenting blood flow, oxygenation and nutrition to tumor cells and other tumor-associated cells, and significantly contributing to tumor progression and metastasis [42]. Our pan-cancer investigation of AFAP1L1 revealed a strong association with endothelial cells. To delve deeper into the role of AFAP1L1 in the tumor microenvironment, we generated murine-specific AFAP1L1 shRNA(shAFAP1L1-Mus) to minimize its influence on human A549 tumor cells. We verified the species specificity and knockdown efficiency of shAFAP1L1-Mus via RT-qPCR assay. In brief, our findings revealed that shAFAP1L1-Mus #1-#2 exhibited robust knockdown efficacy against murine Lewis cells, while shAFAP1L1-Mus #1-#2 demonstrated poor knockdown efficacy against human A549 cells (Additional file 4: Fig. S4A, B). To better comprehend the effect of AFAP1L1 on the tumor microenvironment, we loaded shAFAP1L1-Mus into adeno-associated viruses (AAV) under the control of the endothelial-specific promoter (TIE) (AFAP1L1-ECKD #1-#2) for subsequent in vivo experiments. A cell-derived xenograft (CDX) model of non-small cell lung cancer (NSCLC) was established by subcutaneously implanting A549 cells in 5-week-old BALB/c nude mice. The shRNA was administered daily for 7 days once the tumor expanded to 100mm^3^, and the tumor was excised 15 days later. Our results demonstrated that AFAP1L1 knockdown led to a significant suppression of tumor weight and volume compared to the normal control group (Fig. 6A–C). The tumor volumes in the AFAP1L1 knockout group and the normal control group were 767.723 ± 133.645mm3, 738.288 ± 75.122mm3 and 1832.006 ± 106.33 mm3, respectively (Fig. 6B). The tumor weight of the AFAP1L1 knockdown group and the normal control group were 0.93525 ± 0.099 g, 0.853 ± 0.106 g and 1.93925 ± 0.095 g, respectively (Fig. 6C). Subsequently, to determine the impact of AFAP1L1 on tumor angiogenesis, we stained tumor sections with CD31. Our results demonstrated a substantial reduction in the number of blood vessels in the AFAP1L1 knockdown group compared to the normal control group (Fig. 6D, E). In conclusion, these data imply that AFAP1L1 knockdown suppresses tumor growth by inhibiting neo-angiogenesis.

### AFAP1L1 regulates ocular neovascularization by regulating Vascular Tip cells in vivo

**Fig. 7:**
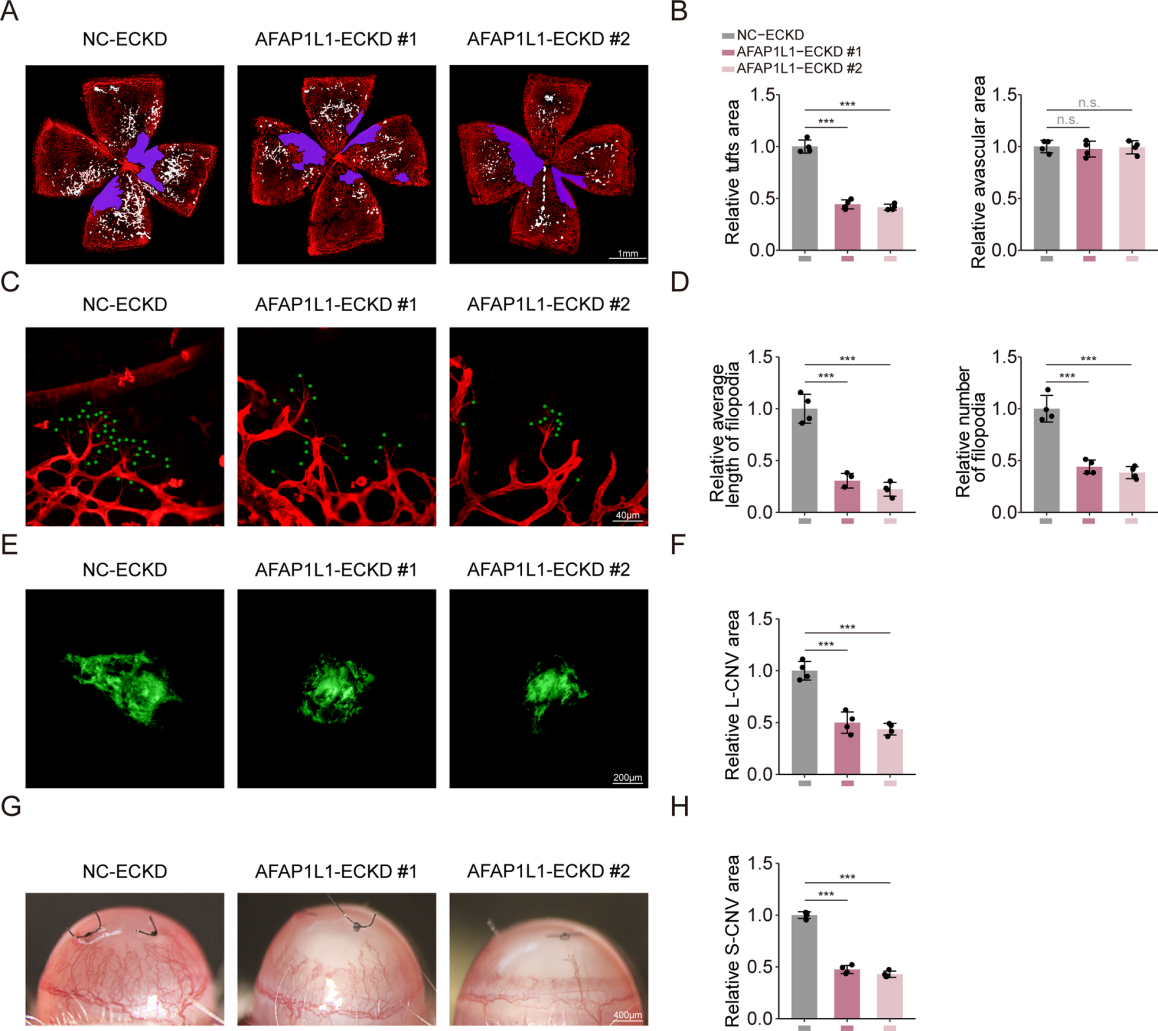
AFAP1L1 knockdown promotes ocular neovascularization in vivo. **A**–**D** IsoB4 staining of whole-mount retinas from OIR mice at P17 intravitreously injected with NC-ECKD, AFAP1L1-ECKD #1 or AFAP1L1-ECKD #2 (Scale bar: 1 mm) (n = 4 mice for each group); the purple area indicates avascular area, and the white area indicates NVTs (**A**). Quantification of neovascular tuft area and avascular area in OIR mice (**B**). The red areas highlighted vascular area especially filopodia in the anterior retina of the nonperfusion area (**C**). Quantification of the number and length of filopodia (green asterisk) respectively. (Scale bar: 40 μm) (n = 4 mice for each group) (**D**). **E**–**F** IsoB4 staining of whole-mount retinas from L-CNV mice intravitreously injected with NC-ECKD, AFAP1L1-ECKD #1 or AFAP1L1-ECKD #2. (n = 4 mice for each group) (**E**). Quantification of size of CNV lesions in L-CNV mice (Scale bar: 200 μm) (**F**). **G**–**H** All the cornea samples of S-CNV mice subconjunctivally injected with NC-ECKD, AFAP1L1-ECKD #1 or AFAP1L1-ECKD #2 were collected for vascular visualization by slit lamp. (n = 4 mice for each group) (**G**). Quantification of size of CNV lesions in S-CNV mice **H** (Scale bar: 400 μm). P values were calculated by one-way ANOVA with Bonferroni's post hoc test **B**, **D**, **F**, **H**. Error bars represent the mean ± SD. *: p-value < 0.05; **: p-value < 0.01; ***: p-value < 0.001; n.s.: no significance

The eye serves as a dependable and convenient platform to investigate the intricate mechanisms of angiogenesis and identify targets for therapeutic interventions aimed at treating pathologic neovascularization [43]. In the OIR model, the cluster of nascent blood vessels generated during retinal vascular regeneration closely mimics the pathological process of retinal angiogenesis, resembling the condition of hypoxic-ischemic retinopathy. In the L-CNV model, neovascularization induced by laser penetrates the sub-retinal pigment epithelium (sub-RPE) or the subretinal space via a rupture in the Bruch membrane, primarily replicating the pathological process of choroid angiogenesis in patients afflicted with wet age-related macular degeneration. In the S-CNV model, neovascularization induced by sutures infiltrates an otherwise transparent and devoid-of-blood-vessels cornea, closely resembling the development of neovascularization during corneal surgery, particularly in the context of corneal transplantation [39]. Therefore, we selected the aforementioned models to assess the role of AFAP1L1 in retinal pathologic angiogenesis and choroidal pathologic angiogenesis. In the OIR model, the IsoB4-stained OIR retina of the AFAP1L1 endothelial specific knockdown group (AFAP1L1-ECKD #1-#2) demonstrated significantly reduced retinal neovascularization and a smaller area of neovascularization plexus when compared to the normal control group (Fig. 7A, B). However, the non-vascular areas of the retina remained unaffected (Fig. 7A, B). Moreover, in the AFAP1L1 endothelial specific knockdown group, the number and length of filopodia in the anterior retina of the nonperfusion area were also substantially diminished (Fig. 7C, D), indicating that AFAP1L1 appears to regulate OIR neovascularization by governing tip cell behavior. In the L-CNV model, we observed a similar trend, with IsoB4-stained area demonstrating smaller L-CNV formation in the AFAP1L1 endothelial specific knockdown group when compared to normal controls (Fig. 7E, F). In the S-CNV model, diminished neovascularization was observed in the cornea of the AFAP1L1 endothelial specific knockdown group through the utilization of slit-lamp microscopy (Fig. 7G, H). Based on the previous bioinformatics analysis and the localization of AFAP1L1 in retinal vascular tip cells, we believe that AFAP1L1 can affect the occurrence and development of ocular pathologic neovascularization by regulating the behavior of the tip cells.

### Hypoxia promotes AFAP1L1 expression through HIF-1α

**Fig. 8:**
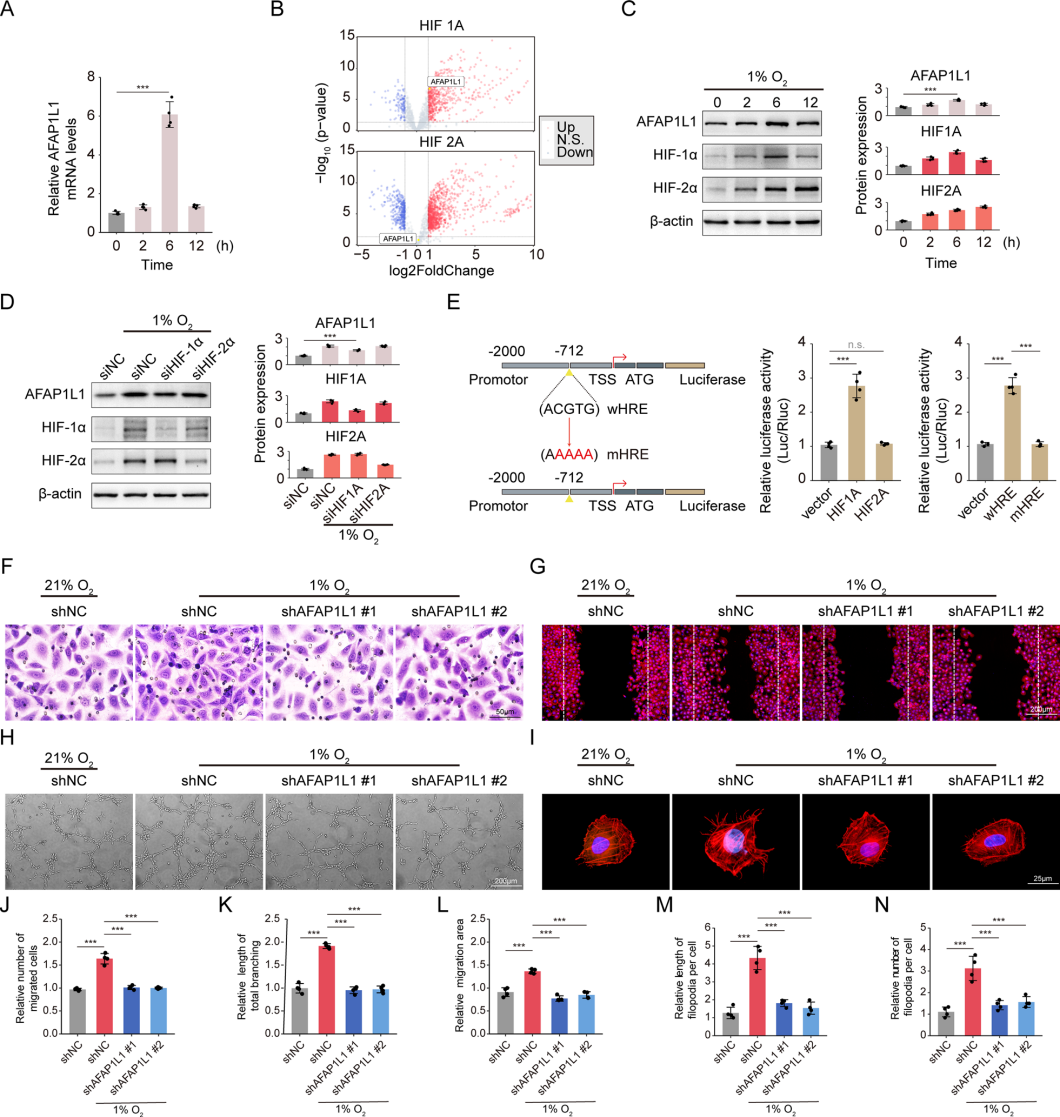
HIF-1α directly suppresses AFAP1L1 transcription under hypoxia. **A** AFAP1L1mRNA levels in HUVEC cells exposed to hypoxia (1% O2) for the indicated times. (n = 4 independent experiments). **B** Volcano plots showed the correlation between AFAP1L1 and HIF-1α or HIF-2α. **C** Western blot analysis of AFAP1L1, HIF-1α, HIF-2α protein levels in HUVEC cells exposed to hypoxia (1% O2) for the indicated times. Densitometric quantitation of western blot band intensity shown in C. Results are presented as mean ± SEM, statistical analyses were performed using one-way ANOVA with Bonferroni's post hoc test. (n = 4 independent experiments). **D** Western blot analysis of AFAP1L1, HIF-1α, HIF-2α and β-actin protein levels in HUVEC cells left untreated or exposed to hypoxia (1% O2) for 24 h as well as transfected with siNC, siHIF-1α or siHIF-2α. Densitometric quantitation of western blot band intensity shown in D. Results are presented as mean ± SEM, statistical analyses were performed using one-way ANOVA with Bonferroni's post hoc test. (n = 4 independent experiments). **E** Schematic diagram depicting the human AFAP1L1 promoter with the presence of hypoxia response element (HRE) sites from the JASPAR database and constructed mutant HRE sites (left panel) (TSS: transcription start site; ATG: initiating methionine codon). Luciferase reporter assay for AFAP1L1 promoter activity in HEK293T cells transfected with control vector, HIF-1α overexpression or HIF-2α overexpression plasmids (n = 4 independent experiments) (middle panel). Luciferase reporter assay for AFAP1L1 promoter activity in HEK293T cells following transfection of wHRE or mHRE luciferase reporter plasmids under HIF-1α overexpression (right panel). (n = 4 independent experiments). P values were calculated by one-way ANOVA with Bonferroni's post hoc test (**A**, **C**, **F**). Error bars represent the mean ± SD. HUVECs are transfected with shNC, shAFAP1L1 #1 or shAFAP1L1 #2 and then exposed to hypoxia (1% O2) for 24 h or left untreated. **F** Transwell assays of HUVECs under hypoxia (1% O2) or simultaneously transfected with shRNA. Scale bar: 50 µm. **G** Wound scratching assays of HUVECs under hypoxia (1% O2) or simultaneously transfected with shRNA. Scale bar: 200 µm. [rhodamine-conjugated phalloidin: red (to visualize the actin cytoskeleton); DAPI: blue (to visualize nuclei)]. **H** Tube formation Assay of HUVECs under hypoxia (1% O2) or simultaneously transfected with shRNA. Scale bar: 200 µm. **I** Rhodamine-phalloidin staining reveals the actin cytoskeleton and filopodia of HUVECs under hypoxia (1% O2) or simultaneously transfected with shRNA (rhodamine-conjugated phalloidin: red; DAPI: blue). Scale bar: 25 µm. **J** Quantification of migrated cells. Results are presented as mean ± SEM, statistical analyses were performed using one-way ANOVA with Bonferroni's post hoc test. (n = 4 per group, data pooled from 4 independent experiments). **K** Quantification of wound area. Results are presented as mean ± SEM, statistical analyses were performed using one-way ANOVA with Bonferroni's post hoc test. (n = 4 per group, data pooled from 4 independent experiments). **L** Quantification of tube formation length. Results are presented as mean ± SEM, statistical analyses were performed using one-way ANOVA with Bonferroni's post hoc test. (n = 4 independent experiments). **M**, **N** Quantification of length and number of filopodia per cell. Results are presented as mean ± SEM, statistical analyses were performed using Kruskal–Wallis with Bonferroni’s post hoc test. (n = 4 independent experiments). *: p-value < 0.05; **: p-value < 0.01; ***: p-value < 0.001; n.s.: no significance

Previous research has indicated that the hypoxia signal is a shared characteristic of tumorigenesis [44], retinopathy of prematurity [45], and wet age-related macular degeneration [46], and is the leading factor in pathological angiogenesis. The anomalous activation of endothelial cells in a hypoxic environment plays a critical role in the formation and progression of tumor blood vessels and ocular pathological neovascularization. Given that AFAP1L1 expression is known to be positively correlated with hypoxia signaling in most cancer types (Fig. 5D), it prompted us to explore whether hypoxia could have an impact on AFAP1L1 expression. Notably, RT-qPCR showed that AFAP1L1 mRNA level was up-regulated in endothelial cells exposed to hypoxia (1% O2) (Fig. 8A), and western blot analysis showed that AFAP1L1 protein expression reached the highest level after 6 h of hypoxia, and then gradually decreased (Fig. 8C). Hypoxia-inducible factors (HIFs), particularly HIF-1α and HIF-2α, are the main drivers of hypoxia, mediating a wide range of hypoxia-induced transcriptional responses by binding to hypoxic response elements (HREs), including the 5'-ACGTG-3' or 5'-CCGTG-3' variants [47, 48]. To ascertain the primary hypoxic regulator of AFAP1L1 between HIF-1α and HIF-2α, we acquired publicly available data from the GEO database (GSE98060) [49]. This dataset consists of RNA sequencing analysis performed on HUVECs that were subjected to HIF-1α and HIF-2α overexpression. Subsequently, we conducted volcano visualization on the obtained data. The findings revealed a significant upregulation of AFAP1L1 mRNA in HUVECs with HIF-1α overexpression (p-value < 0.05, log2FoldChange > 1). Conversely, there was no noteworthy change in AFAP1L1 expression following HIF-2α overexpression (p-value > 0.05, |log2FoldChange|< 1) (Fig. 8B). Subsequent Western blot analysis demonstrated that the expression of HIF-1α protein peaked after 6 h of hypoxia, gradually declining thereafter. Conversely, HIF-2α expression continued to increase (Fig. 8C). These observations led us to hypothesize that HIF-1α may serve as the upstream regulator of AFAP1L1, as both HIF-1α and AFAP1L1 protein expression exhibited a similar pattern under hypoxic conditions (Fig. 8C). To gain deeper insights into the impact of HIF-1α and HIF-2α on AFAP1L1 expression, we employed siRNA technology to selectively suppress the levels of HIF-1α and HIF-2α in HUVECs cells, respectively. Following a 6-h period of hypoxia (1% O2), the expression level of AFAP1L1 was assessed using western blot analysis. The findings revealed that the knockdown of HIF-1α had a significant inhibitory effect on the expression level of AFAP1L1 induced by hypoxia. Conversely, the knockdown of HIF-2α did not exert any discernible influence on the expression level of AFAP1L1 (Fig. 8D).

Through a luciferase reporter gene assay, we confirmed that the transcriptional activity of AFAP1L1 increased in HEK293T cells overexpressing HIF-1α, but not in those overexpressing HIF-2α (Fig. 8E), indicating that HIF-1α is the transcriptional activator of AFAP1L1. To determine if there were any direct interactions between HIF-1α and the HRE regulatory sites, a putative HRE site (ACGTG, relative to TSS, at − 716 to − 712 bp) in the AFAP1L1 promoter region was predicted using the JASPAR database with a relative score cutoff of 0.80 (Fig. 8E). We then transfected wild-type HRE (wHRE) vectors or mutant HRE (mHRE) vectors into HEK293T cells overexpressing HIF-1α, and examined the role of this HRE site in HIF-1α-driven transcriptional regulation by luciferase reporter assay. As expected, under HIF-1α overexpression, luciferase expression was significantly upregulated in reporter plasmids containing wild-type HRE, but not in those containing mutant sequences (Fig. 8E). Taken together, these findings suggest that HIF-1α directly binds to the HRE site in the AFAP1L1 promoter region and promotes transcription during hypoxia.

To investigate the function of AFAP1L1 in HUVECs under anoxic conditions in vitro, we transfected HUVECs with AFAP1L1 knockdown lentiviruses (shAFAP1L1-Homo #1 and #2) or control knockdown lentiviruses (shNC) carrying non-specific sequences. Subsequently, we utilized western blot analysis to detect the knock inefficiency (~ 70% knock inefficiency) (Additional file 4: Fig. S4C). To evaluate the in vitro angiogenic potential of transfected HUVECs under hypoxic conditions (1% O2) over a period of 24 h, assays were conducted including transwell migration, wound healing and tube formation. The results indicated that anoxia significantly increased migration and tube formation capacity of HUVECs, and these effects were reversed by AFAP1L1 knockdown (Fig. 8F-H, J-L). Given that AFAP1L1 is an important member of the actin filament-associated proteins family, it is primarily involved in the regulation of actin cytoskeleton dynamics. Therefore, we investigated the impact of AFAP1L1 on the actin cytoskeleton of vascular endothelial cells. To visualize the actin cytoskeleton, Rhodamine-Phalloidin staining was performed on transfected HUVECs after 24 h of anoxic exposure (1%O2). The findings demonstrated that AFAP1L1 knockdown was capable of reversing hypoxia-induced morphological changes and affecting cytoskeletal structure of HUVECs. To our astonishment, in conjunction with cytoskeletal reorganization, modifications were observed in cellular protrusions, particularly the quantity and length of filopodia, which play a crucial role in directing cells to undergo directed migration and sprouting, were diminished (Fig. 8I, M, N). This outcome was consistent with the observed decrease in the number of filopodia in the anterior retina of mice with AFAP1L1 knockdown. In conclusion, our findings suggest that AFAP1L1 may impact angiogenesis in vitro by regulating the actin cytoskeleton of endothelial cells.

### AFAP1L1 regulates the angiogenic activity of endothelial cells via YAP-DLL4 signal path

**Fig. 9:**
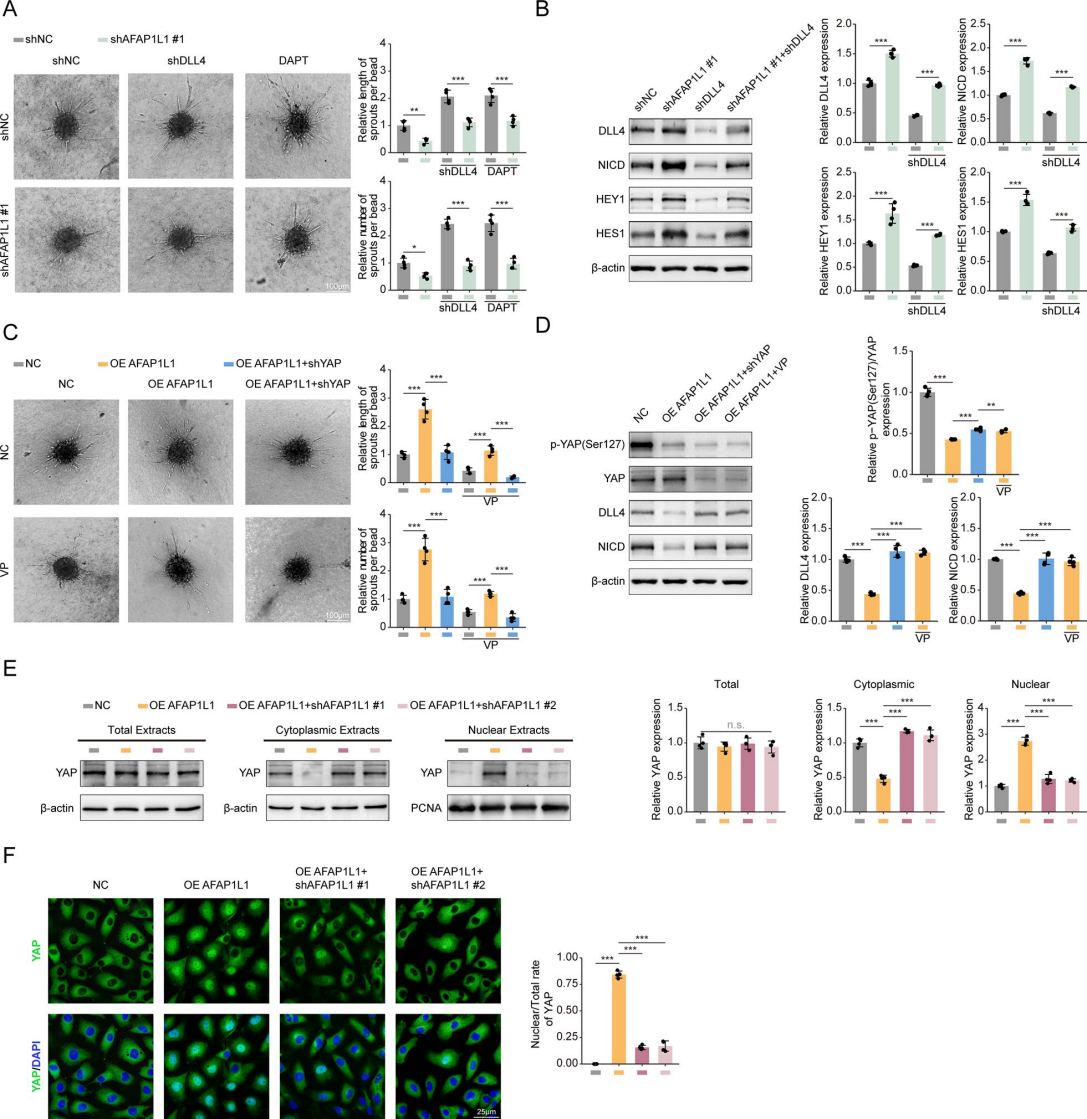
AFAP1L1 regulates the angiogenic activity of ECs via the YAP-DLL4-NOTCH axis. **A** HUVECs were transfected with shNC, shAFAP1L1 #1, shYAP or simultaneously added with DAPT for 24 h. Three-dimensional (3D) Bead Sprouting Assay reveals the in vitro sprouting capabilities of HUVECs under different treatments. Scale bar: 100 µm. Quantification of length and number of sprouts per bead. Results are presented as mean ± SEM, statistical analyses were performed using one-way ANOVA with Bonferroni's post hoc test. (n = 4 independent experiments). **B** Western blot analysis of DLL4, NICD, HES1, HES1 and β-actin protein levels in HUVEC cells transfected with shNC, shAFAP1L1 #1, shYAP or simultaneously added with DAPT for 24 h. Densitometric quantitation of western blot band intensity shown in B. Results are presented as mean ± SEM, statistical analyses were performed using one-way ANOVA with Bonferroni's post hoc test. (n = 4 independent experiments). **C** HUVECs were transfected with Vector, OE AFAP1L1 #1, shYAP or added with VP for 24 h. Three-dimensional (3D) Bead Sprouting Assay reveals the in vitro sprouting capabilities of HUVECs under different treatments. Scale bar: 100 µm. Quantification of length and number of sprouts per bead. Results are presented as mean ± SEM, statistical analyses were performed using one-way ANOVA with Bonferroni's post hoc test. (n = 4 independent experiments). **D** Western blot analysis of p-YAP(Ser127), YAP, DLL4, NICD and β-actin protein levels in HUVEC cells transfected with Vector, OE AFAP1L1, shYAP or simultaneously added with VP for 24 h. Densitometric quantitation of western blot band intensity shown in D. Results are presented as mean ± SEM, statistical analyses were performed using one-way ANOVA with Bonferroni's post hoc test. (n = 4 independent experiments). **E** HUVECs were transfected with Vector, OE AFAP1L1 or simultaneously transfected with shAFAP1L1 #1 or shAFAP1L1 #2. Total, cytoplasmic, nuclear extracts from the resulting cells are analyzed by western blot for YAP expression. Densitometric quantitation of western blot band intensity shown in E Results are presented as mean ± SEM, statistical analyses were performed using one-way ANOVA with Bonferroni's post hoc test. (n = 4 independent experiments). **F** Localization of YAP is demonstrated by immunofluorescence. Scale bar, 25 μm. Quantification of the rate of Nuclear/Total YAP fluorescence. Results are presented as mean ± SEM, statistical analyses were performed using Kruskal–Wallis with Bonferroni's post hoc test. (n = 4 independent experiments). *: p-value < 0.05; **: p-value < 0.01; ***: p-value < 0.001; n.s.: no significance

We then investigated the downstream mechanism of AFAP1L1 promoting angiogenesis. As mentioned earlier, we observed that AFAP1L1 colocalized with DLL4, a tip cell marker in the front of the mouse retina (Fig. 4H), and wondered whether AFAP1L1 could influence tip cell behavior through DLL4. The sprouting of HUVECs in three-dimensional environment was analyzed based on the ball germination test. The model promotes intercellular signaling between endothelial cells and better simulates the sprouting behavior of tip cells in vivo. The results of 3D bead sprouting assay showed that the length and number of sprouts of HUVECs coated beads transfected with shAFAP1L1 decreased (Fig. 9A). Subsequently, we constructed DLL4 knockdown lentivirus (shDLL4) and transfected HUVECs either alone or co-transfected with shAFAP1L1. We then conducted 3D sprouting experiments and found that HUVECs with downregulated DLL4 expression exhibited a significant increase in sprouting ability, which also could rescue the sprouting defects induced by shAFAP1L1 (Fig. 9A). These results suggest that the decreased sprouting ability caused by shAFAP1L1 may be attributed to an upregulation of Dll4 expression. Furthermore, we performed a Western blot analysis to examine the effect of AFAP1L1 on DLL4 expression and observed that the downregulation of AFAP1L1 led to an increase in the expression of DLL4 (Fig. 9B), which was consistent with our 3D sprouting experiment results.

DLL4 is a classical ligand that binds to the NOTCH receptor. Upon cleavage by γ-secretase, the NOTCH receptors release the NOTCH intracellular domain (NICD), which enters the nucleus and binds to the DNA-binding protein CSL, thereby activating NOTCH downstream targets such as HES1 and HEY1[50, 51]. Therefore, we investigated the influence of AFAP1L1 on the NICD and its downstream targets. Our Western blot results showed that downregulation of AFAP1L1 led to an upregulation of NICD, HES1, and HEY1 expression levels (Fig. 9B). Interestingly, the up-regulation induced by shAFAP1L1 could be inhibited by shDLL4 (Fig. 9B), which also indicated that AFAP1L1 affects NOTCH signaling by regulating DLL4 expression. Moreover, when we inhibited NICD release using the γ-secretase inhibitor N-[N-(3,5-difluorophenacetyl)-l-alanyl]-(S)-phenylglycine t-butyl ester (DAPT), shAFAP1L1-induced sprouting defects were significantly rescued (Fig. 9A). In conclusion, it is suggested that AFAP1L1 mainly triggers a cascade reaction of NOTCH downstream signaling through downregulation of DLL4, thereby affecting the sprouting ability of HUVECs.

To investigate the molecular mechanism underlying the augmentation of DLL4 expression induced by shAFAP1L1, our research focused on the transcriptional coactivator YAP/TAZ. Previous investigations have demonstrated the crucial role of YAP/TAZ as a key regulator in sensing cytoskeletal dynamics. Dephosphorylation of YAP leads to its activation and retention within the nucleus [52, 53]. Furthermore, the activation of YAP and TAZ has been found to downregulate DLL4 expression in endothelial cells, thereby influencing the behavior of tip cells [54]. Consequently, we constructed an overexpression plasmid for AFAP1L1 (referred to as OE AFAP1L1) to transfected HUVECs either alone or in conjunction with shYAP after the overexpression efficiency was verified by western blot analysis (Additional file 4: Fig. S4D). Subsequently, we conducted 3D germination experiments, which revealed a significant increase in the germination ability of HUVECs with upregulated AFAP1L1 expression (Fig. 9B). However, this germination ability was significantly reduced following knockdown of YAP (Fig. 9B). Furthermore, the administration of Verteporfin (VP), a YAP inhibitor, resulted in germination defects in HUVECs and effectively prevented excessive germination induced by AFAP1L1 overexpression. The inhibitory effect was more pronounced upon combined application with shYAP (Fig. 9C). Western blot analysis was employed to assess the expression levels of both total and phosphorylated YAP. The results demonstrated that while the overexpression of AFAP1L1 had no impact on the total YAP protein level, it impeded YAP phosphorylation (Fig. 9D). Moreover, YAP activation led to a reduction in the expression of DLL4 and NICD, which were subsequently restored upon transfection of HUVECs with shYAP or the application of Verteporfin (Fig. 9D). Finally, western blot analysis and immunofluorescence tests confirmed that the upregulation of AFAP1L1 expression activated YAP, causing its translocation from the cytoplasm to the nucleus (Fig. 9E, F). Conversely, the administration of shYAP or Verteporfin resulted in the significant removal of nuclear YAP. This partially reversed the effects of AFAP1L1 overexpression induction (Fig. 9E, F). In conclusion, we have ascertained that AFAP1L1 appears to affect the expression of DLL4 and downstream NICD by influencing the phosphorylation status of YAP and its subcellular localization, consequently modulating the behavior of tip cells.

## Discussion

Angiogenesis constitutes a multifaceted process entailing the harmonization of pro-angiogenic elements and inhibitory agents. In normal circumstances, these two entities maintain a state of dynamic equilibrium. However, when this balance is disrupted, the vascular system becomes activated, giving rise to excessive angiogenesis and pathological neovascularization characterized by structural irregularities and aberrant functionality [55]. These vasculatures exert a significant influence on the onset and progression of tumors and neovascular eye disorders by augmenting the provision of oxygen and nutrients to aberrantly growing solid tissues and enhancing the efficiency of metabolic waste disposal. Notably, aberrant endothelial cell function represents a cardinal feature of pathological angiogenesis, with the principal causative factor being the aberrant secretion of vascular endothelial growth factor (VEGF) induced by various pathological stimuli [56]. In recent years, substantial advancements have been achieved in the development of anti-angiogenic drugs targeting VEGF and its receptor (VEGFRs), which now stand as the primary clinical approach for the treatment of tumors and ocular neovascularization [10–12]. However, the efficacy of these drugs is partly impeded by unfavorable reactions and drug resistance [13, 14]. Thus, it holds paramount significance to thoroughly investigate novel targets and comprehensively grasp their biological functions within endothelial cells to effectively regulate the progression of these diseases.

Previous investigations concerning AFAP1L1 were considerably limited and predominantly concentrated on the realm of tumors, primarily influencing cell invasion through the regulation of cytoskeletal dynamics. For instance, within osteosarcoma cellular lineages, the suppression of AFAP1L1 impeded the formation, cellular adhesion, migration, and infiltration of invasive pseudopods [22]. Within this study, we conducted an extensive analysis of numerous multi-omics cancer data sets from various perspectives to further elucidate the functional significance of AFAP1L1 across a spectrum of malignancies. Based on the outcomes of differential expression analysis, prognostic evaluation, methylation and phosphorylation analysis, and other correlation analyses, we deduce that AFAP1L1 is intricately linked to the occurrence and advancement of diverse cancer types. It assumes the role of a cancer-promoting factor across a range of malignancies, including COAD, STAD, LGG, and others. Reassuringly, our analysis aligns harmoniously with numerous preceding studies. For instance, the investigation conducted by Takahashi et al. unveiled a noteworthy upsurge in AFAP1L1 expression within colorectal cancer tissues. Notably, in conjunction with vinculin, it exhibited localization to the ringed structure of invadopodia, consequently fostering cancer progression [20]. Similarly, Sun et al. reported an elevation in AFAP1L1 expression in gastric cancer tissues. Moreover, they discovered that AFAP1L1 induction facilitated epithelial-mesenchymal transition (EMT) to promote the advancement of gastric cancer by activating CDC42 and ITGA5 signaling pathways, with mediation from VAV2 [18].

Nonetheless, the precise involvement of AFAP1L1 in angiogenesis remains ambiguous. By means of immune infiltration analysis and single-cell data analysis, we observed a high correlation between AFAP1L1 and endothelial cells, particularly in the tip cell population where it exhibited significant enrichment. Functional enrichment analysis disclosed the close association of AFAP1L1 with angiogenesis, primarily influencing vascular sprouting and growth. As it is widely acknowledged, endothelial cells can be classified into three primary subtypes: tip cells, stem cells, and phalanx cells [57–59]. Tip cells assume a pivotal role in the angiogenic sprouting process, forming filamentous pseudopods during heightened migratory states to establish the trajectory of blood vessel growth. Furthermore, they possess the ability to detect and respond to the gradient of VEGF, thereby exerting a vital influence on the number, branching pattern, and orientation of nascent blood vessels [58]. Hence, through comprehensive analysis, we have reached the conclusion that AFAP1L1 exerts an impact on angiogenesis, thereby affecting the occurrence and development of tumors and neovascular eye conditions, primarily by modulating the behavior of tip cells. These deductions were further substantiated by our experimental validation. Initially, we observed co-localization of AFAP1L1 with the endothelial tip cell marker DLL4 in retinal tip cells. Subsequently, within multiple pathological models, we observed that the suppression of AFAP1L1 led to a notable inhibition of angiogenesis. Notably, within the ROP model, we meticulously observed a significant reduction in both the quantity and length of filamentous pseudopodia subsequent to the knockdown of AFAP1L1.

Hypoxic microenvironment is a crucial characteristic of all solid tumors, contributing to the occurrence, progression, and treatment resistance of cancer [60]. Hypoxia assumes a significant pathological determinant, inciting the emergence of neovascularization in diverse neovascular ocular ailments, including retinopathy of prematurity [45] and wet age-related macular degeneration [46]. Hypoxia-inducible factors (HIF) are vital transcription factors that respond to cellular hypoxic conditions. Within this family, HIF-1α and its related isoform, HIF-2α, play essential roles in regulating the expression of numerous genes involved in angiogenesis, such as VEGF and Erythropoietin, which are crucial for vascular development and maintenance [61]. Excitingly, the sequencing data presented by Downes et al. [49] indicate that stable overexpression of HIF-1α, rather than HIF-2α, in HUVECs leads to an upregulation of AFAP1L1 mRNA levels. This implies a potential role for AFAP1L1 as a hypoxia-associated regulatory protein responsive to HIF-1α, modulating endothelial cell function. In this study, we observed an elevation in AFAP1L1 expression in hypoxia-treated HUVECs in vitro. Furthermore, our findings indicate that the upregulation of AFAP1L1 is predominantly mediated by the presence of HIF-1α, as HIF-1α facilitated its transcriptional activation by directly binding to the hypoxia response elements in the AFAP1L1 promoter region. Functional experiments conducted on HUVECs in vitro also revealed that knockdown of AFAP1L1 could reverse the excessive angiogenic capacity induced by hypoxia, further supporting the role of AFAP1L1 as a hypoxia-associated regulatory protein and a pivotal modulator of angiogenesis.

Then, how does AFAP1L1 modulate endothelial cell angiogenesis? Previous investigations have demonstrated that AFAP1L1, functioning as an actin filament-associated protein, can orchestrate cytoskeletal rearrangements, including the regulation of pseudopod formation [15, 22]. Within our study, we have established, for the first time, the role of AFAP1L1 in endothelial cells in regulating the dynamics of the cytoskeleton, particularly in relation to the formation of filopodia. YAP/TAZ is an important protein that can sense the mechanical signals imposed by cytoskeletal changes. Changes in extracellular matrix and cellular stress fibers can affect YAP/TAZ activation and nuclear translocation [52, 53]. We found that after AFAP1L1 knockdown, YAP phosphorylation levels in endothelial cells increased, promoting its inactivation and sequestration in the cytoplasm, while the reduction of nuclear YAP led to increased expression of NOTCH classical ligand DLL4, which subsequently conjugated to the NOTCH receptor to promote downstream signaling cascades. Bioinformatics analysis and immunofluorescence results showed that AFAP1L1 was significantly expressed in vascular endothelial tip cells, and was co-located with the tip cell marker DLL4 in the front retina. The up-regulated expression of DLL4 in single tip cells binds to NOTCH receptors in neighboring stem cells to induce NOTCH signaling activation. The downstream target genes Hes1 and Hey1 can down-regulate the expression of VEGFR2 in tip cells, and subsequently inhibit the expression of vascular endothelial tip cell markers and DLL4. This helps to transform the endothelial tip cells into stem cells at the fusion point of angiogenic buds and inhibit the formation of ectopic buds. This also explains the reduction of neovasculum clusters, the number of vascular tip cells and the length of filamentopodia in the responder region after AFAP1L1 knockout in the mouse ROP model. Taken together, we conclude that AFAP1L1 may regulate endothelial cell angiogenesis through the YAP-Dll4-NOTCH axis.

In recent years, gene therapy has emerged as a pivotal research domain in clinical transformation. Notably, investigations centered around AAV as the principal gene delivery vector have been extensively executed within clinical trials, which encompass an array of evaluations, spanning the immunogenicity, toxicity, and persistence characteristics of this vector [62]. Within our study, AFAP1L1 has been identified as a prospective therapeutic target for the modulation of pathological neovascularization. Furthermore, the efficacy of AAV incorporating AFAP1L1 in addressing neoplastic and neovascular ocular disorders has been substantiated through murine experimentation. Our forthcoming inquiry will appraise the in vitro and in vivo toxicity profiles and metabolic behaviors of this delivery modality, thereby fostering its clinical translatability. Additionally, our investigation has unveiled that AFAP1L1, as a hypoxia-related regulatory protein, is regulated by HIF-1α and affects the angiogenesis of endothelial tip cells through the YAP-Dll4-NOTCH signaling pathway. Although DAPT (one inhibitor of NOTCH intracellular domain) have been investigated within the realm of angiogenesis [63], pertinent endeavors concerning safety and pharmacokinetics in the context of neovascular ocular disorders remain conspicuously absent. Subsequently, we shall supplement these investigations to facilitate their translation to clinical realms. However, there are still some limitations in this study, such as although we have revealed the regulatory role of AFAPL1L in endothelial tip cells through above-mentioned signaling pathway in vitro, the exact mechanism by which AFAP1L1 affects tip cell specification is not yet clear. Furthermore. the large gap between in vitro and in vivo environment culture conditions of cells, especially oxygen concentration, which cannot fully reflect the biological function of AFAP1L1 in vivo. In the future, relevant primary cells will be extracted and related in vitro experiments will be conducted under conditions closer to physiological oxygen concentration, and then AFAP1L1 endothelium-specific gene knockout mice were constructed to conduct related in vivo experiments. These future works are more conducive to understanding the role of AFAP1L1 in vivo, provide a more solid and reliable basis for promoting gene therapies targeted against AFAP1L1, and ultimately realize the clinical transformation of AFAP1L1 in the treatment of tumors and ocular pathologic angiogenesis.

### Supplementary Information

**Additional file 1: **Figure S1. Flowchart presenting the steps of integrated bioinformatic analysis. The flowchart outlines the specific analysis methods employed in pan-cancer analysis and single-cell data analysis, their respective objectives, and the relevant R packages utilized.

**Additional file 2: Figure S2.** Analysis of protein phosphorylation of AFAP1L1 in human cancers. The protein phosphorylation of AFAP1L1 in several cancers using the UALCAN web tool with CPTAC dataset. The differences in AFAP1L1 phosphoprotein (including phosphorylation sites S747, S745, S329, S98, S712, S296, and S714) between normal tissue and primary tissue was visualized by box plots. GBM **A**–**D**, LUAD **E**–**G**, HCC **H**–**J**, ovarian cancer **K**–**M**, clear cell RCC **N**–**Q**, and PAAD **R**–**T**. P-value was calculated by unpaired t-test. *: p-value < 0.05; **: p-value <0.01; ***: p-value <0.001; n.s.: no significance.

**Additional file 3: Figure S3. **The tissue cell type classification of AFAP1L1 from the HPA dataset. **A** The core cell type table displays the enrichment scores of AFAP1L1 in eight cell types found in many tissues (the enriched cell type is indicated in purple). **B**–**M** The plots show the enrichment scores for AFAP1L1 in each cell type, in each tissue. An enlarged and bolded circle indicates classification of AFAP1L1 as cell type enriched in the corresponding cell type.

**Additional file 4: Figure S4.** The efficiency of shRNA lentivirus and plasmids was evaluated by qRT-PCR and western blot analysis. **A** QRT-PCR analyses of AFAP1L1 mRNA expression in Lewis cell lines after stable knockdown of AFAP1L1 by shRNA lentiviral transfection. Quantification of the knockdown efficiency of lentiviral shRNA against AFAP1L1. Results are presented as mean ± SEM, statistical analyses were performed using one-way ANOVA with Bonferroni's post hoc test. (n=4 independent experiments). **B** QRT-PCR analyses of AFAP1L1 mRNA expression in A549 cell lines after stable knockdown of AFAP1L1 by shRNA lentiviral transfection. Quantification of the knockdown efficiency of lentiviral shRNA against AFAP1L1. Results are presented as mean ± SEM, statistical analyses were performed using one-way ANOVA with Bonferroni's post hoc test. (n=4 independent experiments). **C** Western blot analyses of AFAP1L1 and β-actin protein expression in HUVEC cells after stable knockdown of AFAP1L1 by shRNA lentiviral transfection. Densitometric quantitation of western blot band intensity shown in C. Results are presented as mean ± SEM, statistical analyses were performed using one-way ANOVA with Bonferroni's post hoc test. (n=4 independent experiments). **D** Western blot analyses of AFAP1L1 and β-actin protein expression in HUVEC cells after overexpression of AFAP1L1 by plasmids transfection. Densitometric quantitation of western blot band intensity shown in D. Results are presented as mean ± SEM, statistical analyses were performed using two-tailed student's t-test. (n=4 independent experiments). *: p-value < 0.05; **: p-value <0.01; ***: p-value <0.001; n.s.: no significance.

**Additional file 5: Table S1.** The abbreviations and corresponding full names of various cancers.

**Additional file 6: Table S2.** The target sequence of plasmid and constructed virus

## Data Availability

Source data and reagents are available from the corresponding author upon reasonable request.
