# Supplementary figures and images for: Hypoxia-induced AFAP1L1 regulates pathological neovascularization via the YAP-DLL4-NOTCH axis
Source: J Transl Med. 2023 Sep 22;21:651. doi: 10.1186/s12967-023-04503-x (PMC10515434; doi:10.1186/s12967-023-04503-x)

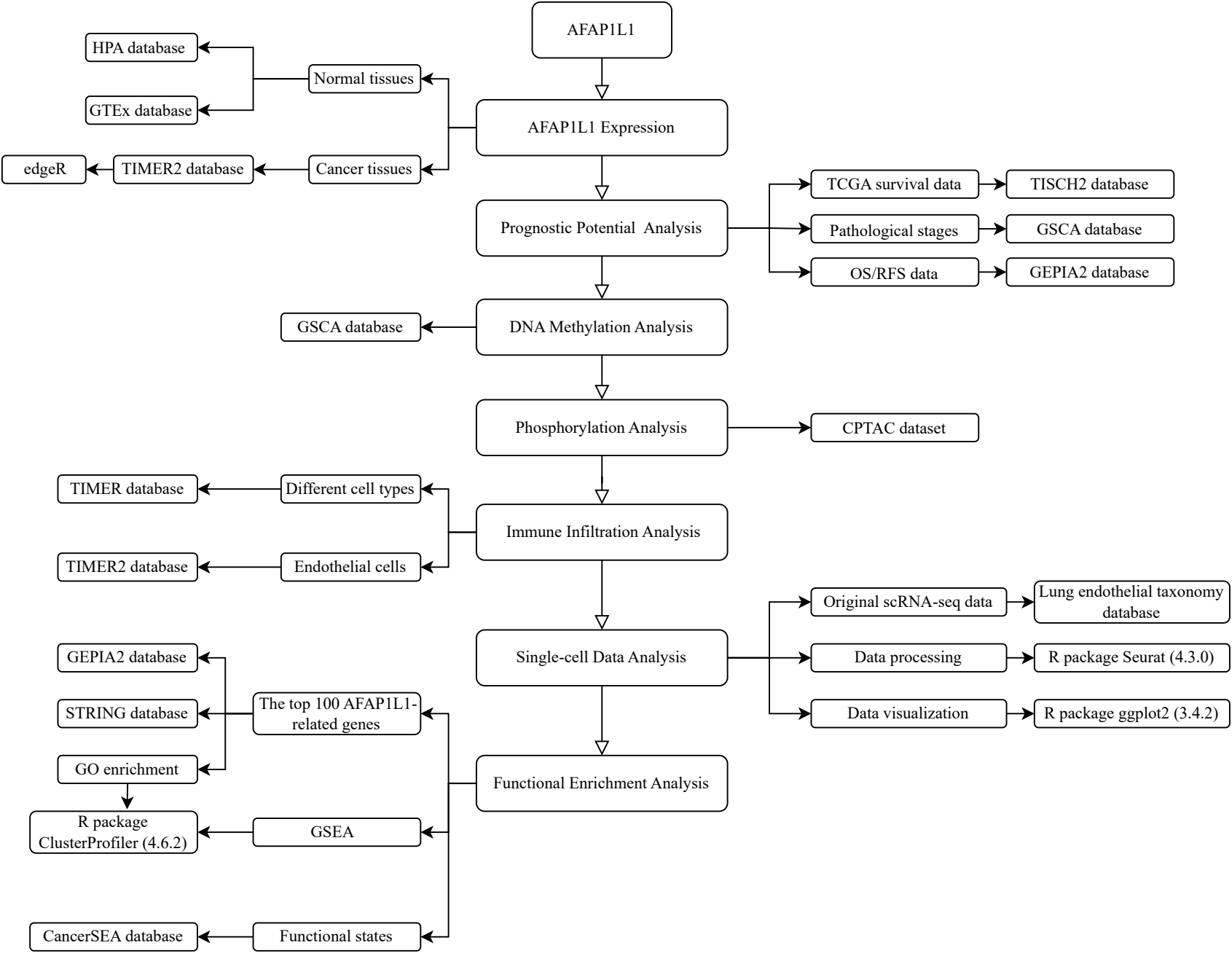

Supplement: Supplementary file 1 — Additional file 1: Figure S1. Flowchart presenting the steps of integrated bioinformatic analysis. The flowchart outlines the specific analysis methods employed in pan-cancer analysis and single-cell data analysis, their respective objectives, and the relevant R packages utilized. [file 12967_2023_4503_MOESM1_ESM.pdf]

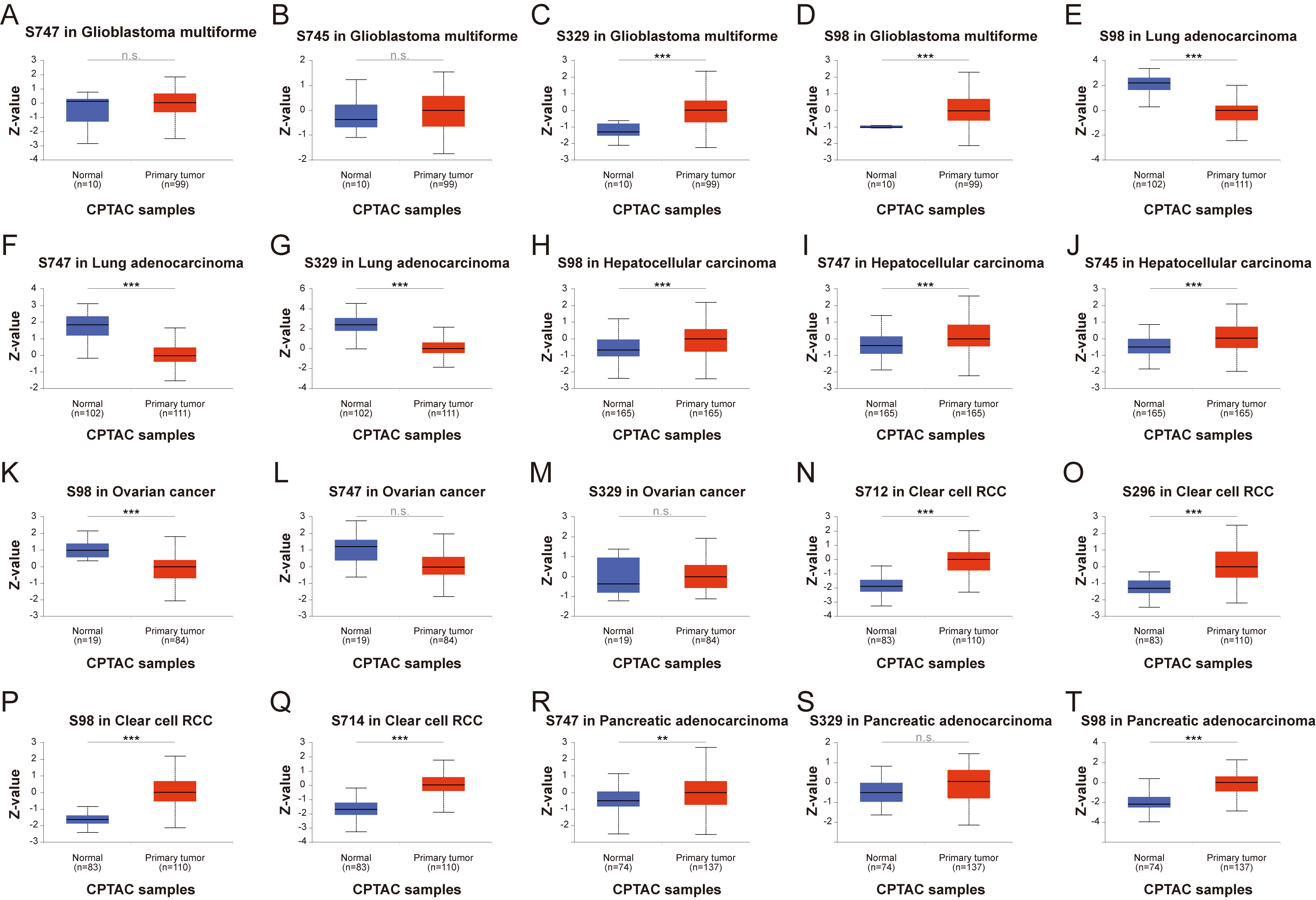

Supplement: Supplementary file 2 — Additional file 2: Figure S2. Analysis of protein phosphorylation of AFAP1L1 in human cancers. The protein phosphorylation of AFAP1L1 in several cancers using the UALCAN web tool with CPTAC dataset. The differences in AFAP1L1 phosphoprotein (including phosphorylation sites S747, S745, S329, S98, S712, S296, and S714) between normal tissue and primary tissue was visualized by box plots. GBM A–D, LUAD E–G, HCC H–J, ovarian cancer K–M, clear cell RCC N–Q, and PAAD R–T. P-value was calculated by unpaired t-test. *: p-value < 0.05; **: p-value <0.01; ***: p-value <0.001; n.s.: no significance. [file 12967_2023_4503_MOESM2_ESM.tif]

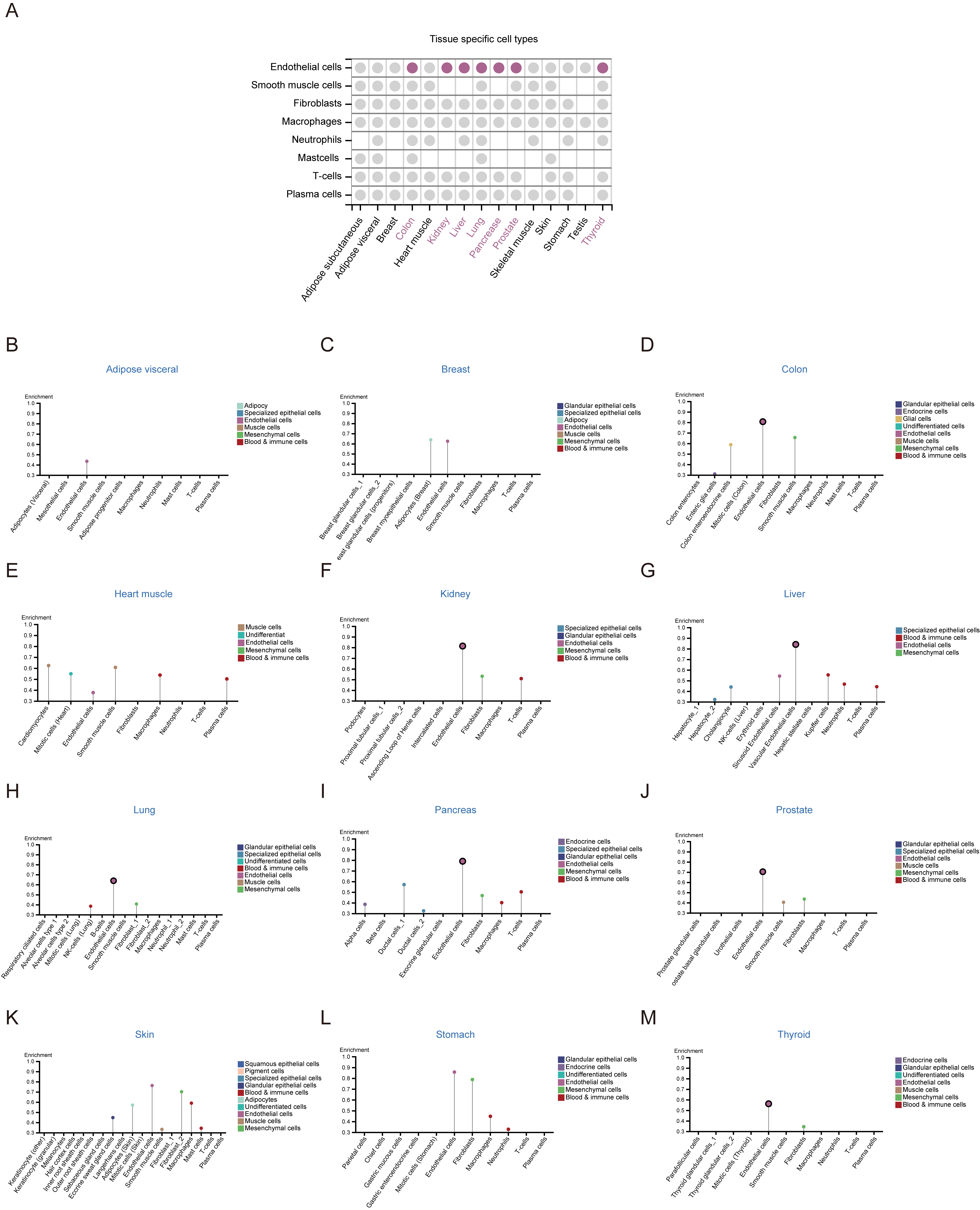

Supplement: Supplementary file 3 — Additional file 3: Figure S3. The tissue cell type classification of AFAP1L1 from the HPA dataset. A The core cell type table displays the enrichment scores of AFAP1L1 in eight cell types found in many tissues (the enriched cell type is indicated in purple). B–M The plots show the enrichment scores for AFAP1L1 in each cell type, in each tissue. An enlarged and bolded circle indicates classification of AFAP1L1 as cell type enriched in the corresponding cell type. [file 12967_2023_4503_MOESM3_ESM.tif]

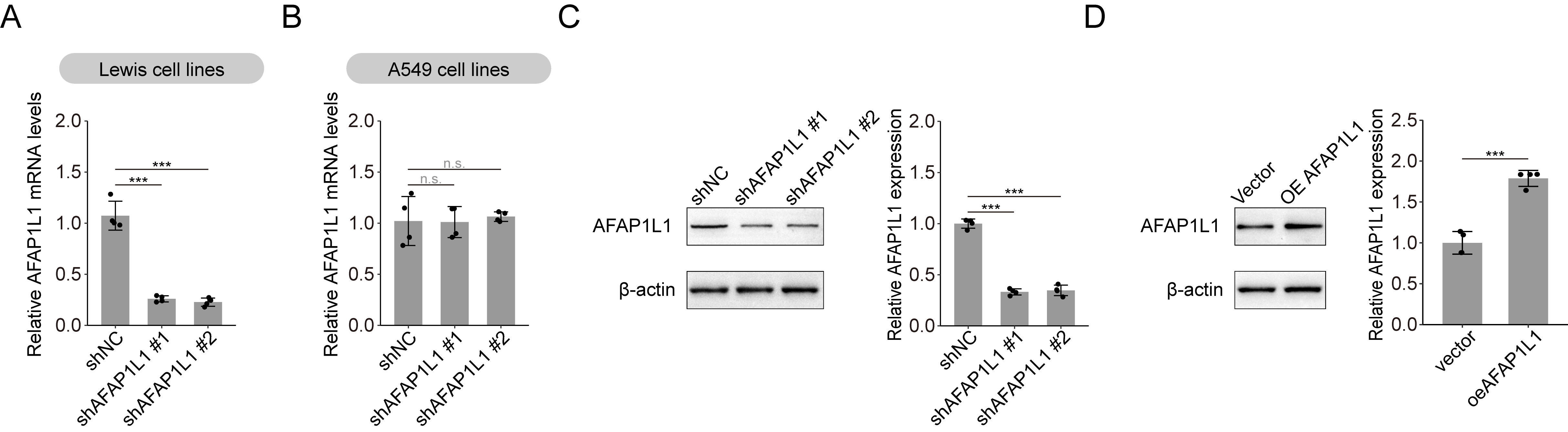

Supplement: Supplementary file 4 — Additional file 4: Figure S4. The efficiency of shRNA lentivirus and plasmids was evaluated by qRT-PCR and western blot analysis. A QRT-PCR analyses of AFAP1L1 mRNA expression in Lewis cell lines after stable knockdown of AFAP1L1 by shRNA lentiviral transfection. Quantification of the knockdown efficiency of lentiviral shRNA against AFAP1L1. Results are presented as mean ± SEM, statistical analyses were performed using one-way ANOVA with Bonferroni's post hoc test. (n=4 independent experiments). B QRT-PCR analyses of AFAP1L1 mRNA expression in A549 cell lines after stable knockdown of AFAP1L1 by shRNA lentiviral transfection. Quantification of the knockdown efficiency of lentiviral shRNA against AFAP1L1. Results are presented as mean ± SEM, statistical analyses were performed using one-way ANOVA with Bonferroni's post hoc test. (n=4 independent experiments). C Western blot analyses of AFAP1L1 and β-actin protein expression in HUVEC cells after stable knockdown of AFAP1L1 by shRNA lentiviral transfection. Densitometric quantitation of western blot band intensity shown in C. Results are presented as mean ± SEM, statistical analyses were performed using one-way ANOVA with Bonferroni's post hoc test. (n=4 independent experiments). D Western blot analyses of AFAP1L1 and β-actin protein expression in HUVEC cells after overexpression of AFAP1L1 by plasmids transfection. Densitometric quantitation of western blot band intensity shown in D. Results are presented as mean ± SEM, statistical analyses were performed using two-tailed student's t-test. (n=4 independent experiments). *: p-value < 0.05; **: p-value <0.01; ***: p-value <0.001; n.s.: no significance. [file 12967_2023_4503_MOESM4_ESM.tif]
