# Supplementary material for: Hypoxia-induced AFAP1L1 regulates pathological neovascularization via the YAP-DLL4-NOTCH axis
Source: J Transl Med. 2023 Sep 22;21:651. doi: 10.1186/s12967-023-04503-x (PMC10515434; doi:10.1186/s12967-023-04503-x)
Supplement: Supplementary file 6 — Additional file 6: Table S2. The target sequence of plasmid and constructed virus [file 12967_2023_4503_MOESM6_ESM.docx]

Table S2 The target sequence of plasmid and constructed virus.

| Gene | SS Sequence | AS Sequence |
| --- | --- | --- |
| AFAP1L1-ECKD #1 | CCAAGACACUACAAAUAAATT | UUUAUUUGUAGUGUCUUGGTT |
| AFAP1L1-ECKD #2 | GGAAGUCUCUUUCCUGUAUTT | AUACAGGAAAGAGACUUCCTT |
| shAFAP1L1-Homo #1 | CGGAGGUACUUGGUAGAAATT | UUUCUACCAAGUACCUCCGTT |
| shAFAP1L1-Homo #2 | GUGGGUGACAACUGUUCUATT | UAGAACAGUUGUCACCCACTT |
| shAFAP1L1-Mus #1 | CCAAGACACUACAAAUAAATT | UUUAUUUGUAGUGUCUUGGTT |
| shAFAP1L1-Mus #2 | GGAAGUCUCUUUCCUGUAUTT | AUACAGGAAAGAGACUUCCTT |
| shYAP-Homo | GGTCAGAGATACTTCTTAA | TTAAGAAGTATCTCTGACC |
| shDLL4-Homo | CAGATGCACTCATCAGCAA | TTGCTGATGAGTGCATCTG |
| siHIF-1α-Homo | GGGAUUAACUCAGUUUGAATT | UUCAAACUGAGUUAAUCCCTT |
| siHIF-2α-Homo | GGAGCUAACAGGACAUAGUTT | ACUAUGUCCUGUUAGCUCCTT |
